# Introduced species shed friends as well as enemies

**DOI:** 10.1038/s41598-024-61788-8

**Published:** 2024-05-15

**Authors:** Zoe A. Xirocostas, Jeff Ollerton, Begoña Peco, Eve Slavich, Stephen P. Bonser, Meelis Pärtel, S. Raghu, Angela T. Moles

**Affiliations:** 1 Evolution and Ecology Research Centre, School of Biological, Earth and Environmental Sciences, UNSW Sydney, Sydney, NSW 2052 Australia; 2 Kunming Institute of Botany, Chinese Academy of Sciences, Kunming, China; 3 Faculty of Arts, Science and Technology, University of Northampton, Northampton, UK; 4 Terrestrial Ecology Group (TEG), Department of Ecology, Institute for Biodiversity and Global Change, Universidad Autónoma de Madrid, 28049 Madrid, Spain; 5 Stats Central, Mark Wainwright Analytical Centre, UNSW Sydney, Sydney, NSW 2052 Australia; 6 Institute of Ecology and Earth Sciences, University of Tartu, J. Liivi 2, 50409 Tartu, Estonia; 7 CSIRO Health & Biosecurity, Brisbane, QLD Australia; 8 Present Address: School of Life Sciences, Faculty of Science, University of Technology Sydney, Sydney, NSW 2007 Australia

**Keywords:** Ecology, Invasive species

## Abstract

Many studies seeking to understand the success of biological invasions focus on species’ escape from negative interactions, such as damage from herbivores, pathogens, or predators in their introduced range (enemy release). However, much less work has been done to assess the possibility that introduced species might shed mutualists such as pollinators, seed dispersers, and mycorrhizae when they are transported to a new range. We ran a cross-continental field study and found that plants were being visited by 2.6 times more potential pollinators with 1.8 times greater richness in their native range than in their introduced range. Understanding both the positive and negative consequences of introduction to a new range can help us predict, monitor, and manage future invasion events.

## Introduction

Throughout history, plants and animals have been introduced to new areas of the globe, either purposefully or accidentally, leading to devastating consequences for natural ecosystems^1–3^. One of the most influential and well-studied ideas about why introduced species are so successful is the Enemy Release Hypothesis^4,5^, which suggests that introduced species escape some of their co-evolved predators, pathogens and herbivores when they move to a new range^4–7^. Release from enemies can allow introduced species to decrease investment in defences, increase their competitive ability, and promote expansion into new ranges^4,8^. However, what is less commonly considered is that plants’ enemies may not be the only thing they leave behind.

**Figure 1:**
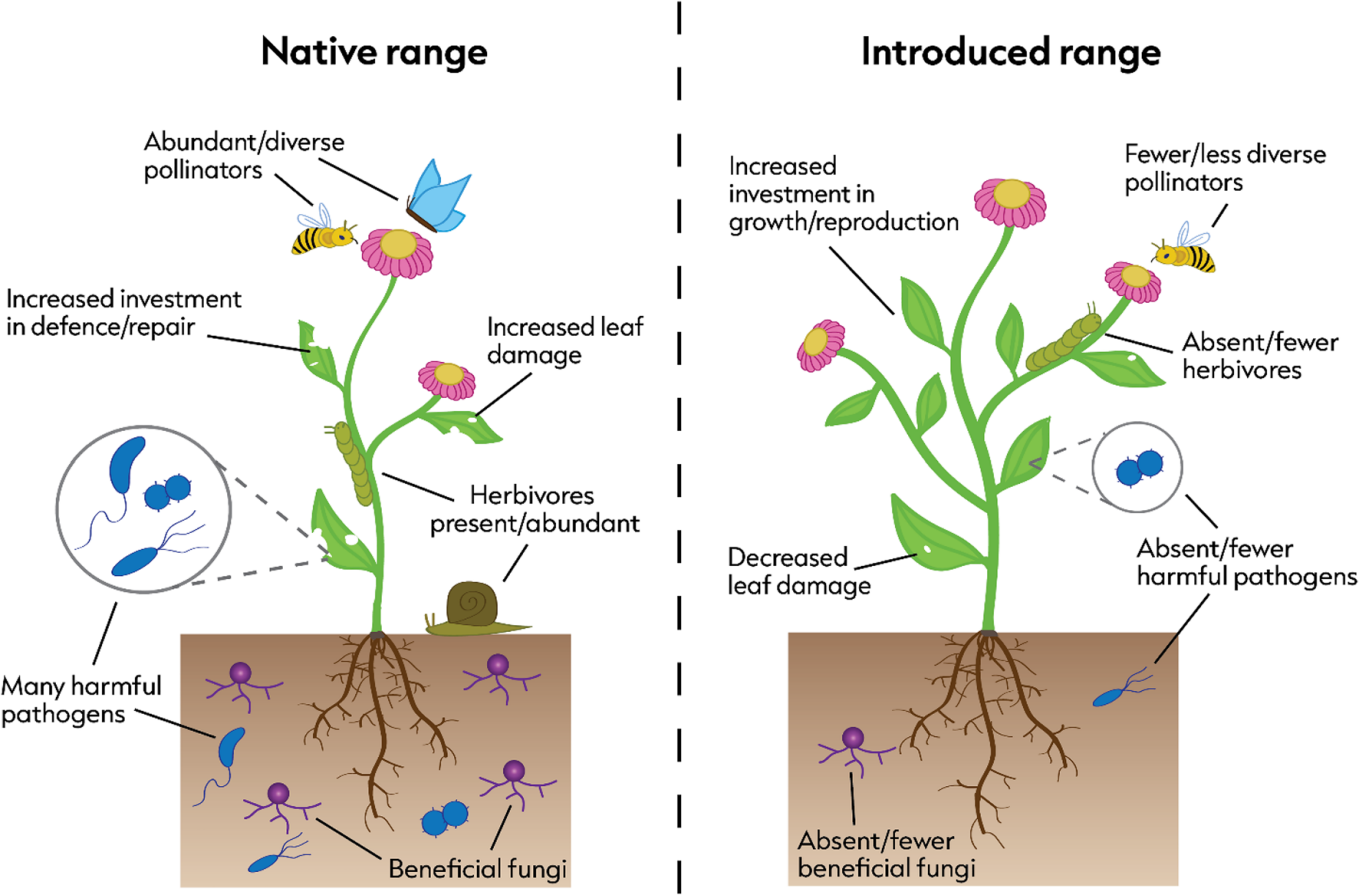
Conceptual illustration of the differences in key interactions (non-exhaustive) explained by the Enemy Release Hypothesis and Missed Mutualist Hypothesis in plants’ native and introduced ranges. In this paper we consider interactions between plants and herbivores/flower visitors only.

In this paper, we test the idea that plant species escape their friends as well as their enemies when introduced to a new environment ^9–11^ (The Missed Mutualist Hypothesis, Fig. 1). Separation from mutualists such as pollinators, seed dispersers and mycorrhizae could decrease plant fitness, generate negative selective pressures, and increase the probability of extinction for introduced populations^12^. Evidence for missed mutualisms is extremely sparse in comparison to enemy release^10^, and the non-random selection of target species coupled with the fact that most studies focus on a single species, could lead to biases when estimating the effect size of missed mutualisms. Here we make the first comprehensive assessment of The Missed Mutualist Hypothesis by assessing pollination, one of the most common and necessary mutualisms in over 87% of angiosperms^13,14^, across ten plant species in nine locations within their native and introduced ranges.

### Far fewer flower visitors

We began by testing the hypothesis that the abundance and taxonomic richness of flower visitors will be lower in the introduced range than in the native range. Interacting with more, or more types of pollinators improves plants’ ability to produce more fruits and set more seeds^15^. Therefore, a decrease in the richness of flower visitors is predicted to have a negative impact on successful reproduction, and ultimately invasion. When a plant species invades, it can be integrated into the interaction-web of generalist pollinators^16,17^. But this uptake of some generalists may not be enough to compensate for the decrease in the richness of flower visitors plants receive in their introduced range. Few studies investigating the impact of range on pollinator richness currently exist^10^ and often compare introduced species with native species occupying the same habitats^16,18–20^. Our biogeographic comparison of species across their native and introduced ranges enables us to detect patterns that may not be as visible when using a community comparative approach^6,21^.

**Figure 2:**
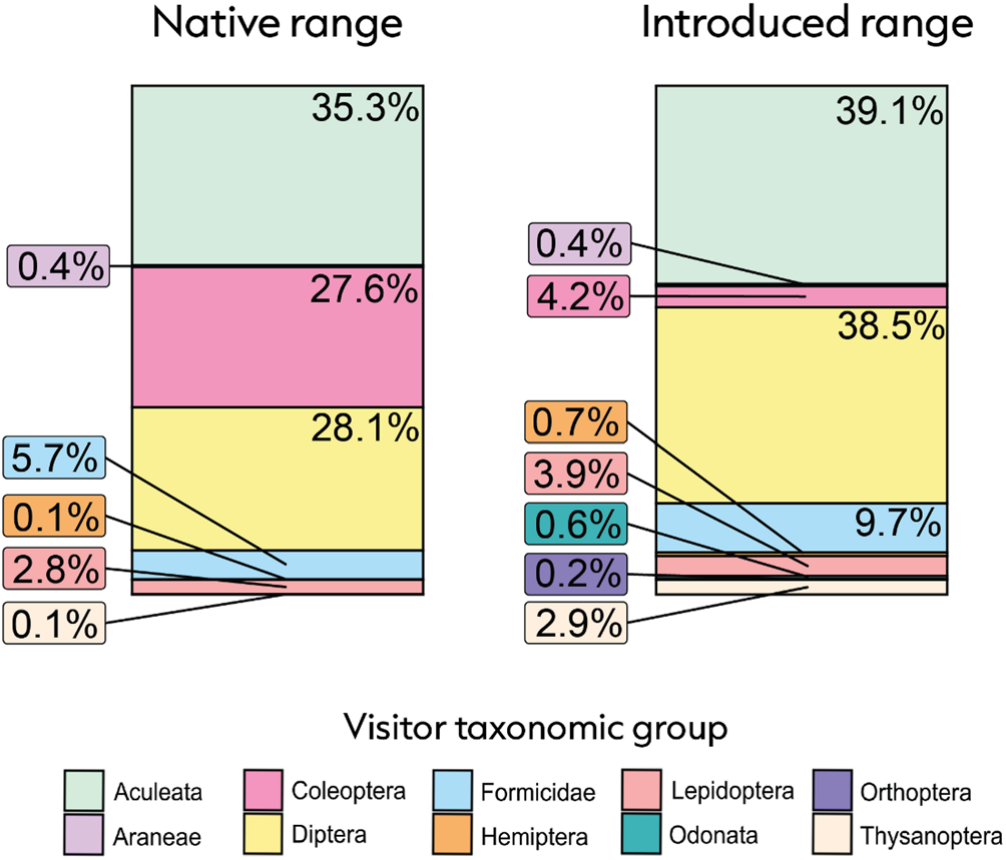
In plants’ native range, we observed 1592 flower visits which were dominated by bees and wasps (Aculeata; 35.3% of interactions), followed by flies (Diptera; 28.1% of interactions), and beetles (Coleoptera; 27.6% of interactions). In the introduced range we observed 1060 interactions which comprised mostly of bees and wasps (39.1% of interactions), flies (38.5% of interactions), and ants (Formicidae; 9.7% of interactions).

**Figure 3:**
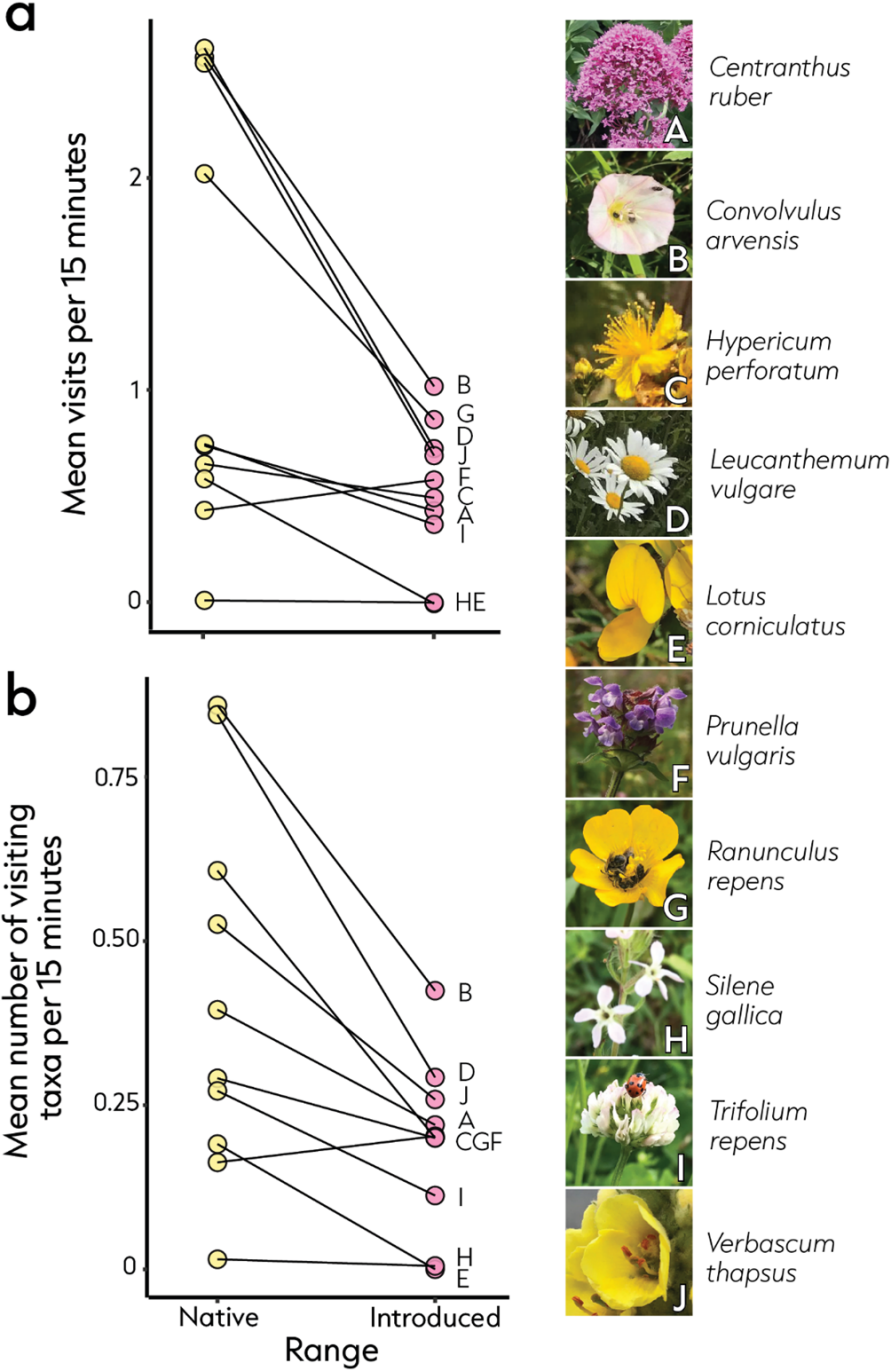
Comparison of (**a**) the mean number of flower visits and (**b**) mean number of visiting taxa per 15-min observation for each species in the native (yellow) and introduced (pink) ranges. To calculate this metric, visits were first divided by the number of floral units per observation before taking means (per species per site) [see Supplementary information for variance across sites and model coefficients/confidence intervals per species]. Images of plant species observed in this study are displayed to the right of both graphs accompanied by letters A-J which correspond to letters displayed on the graphs (all images credited to Z Xirocostas).

**Figure 4:**
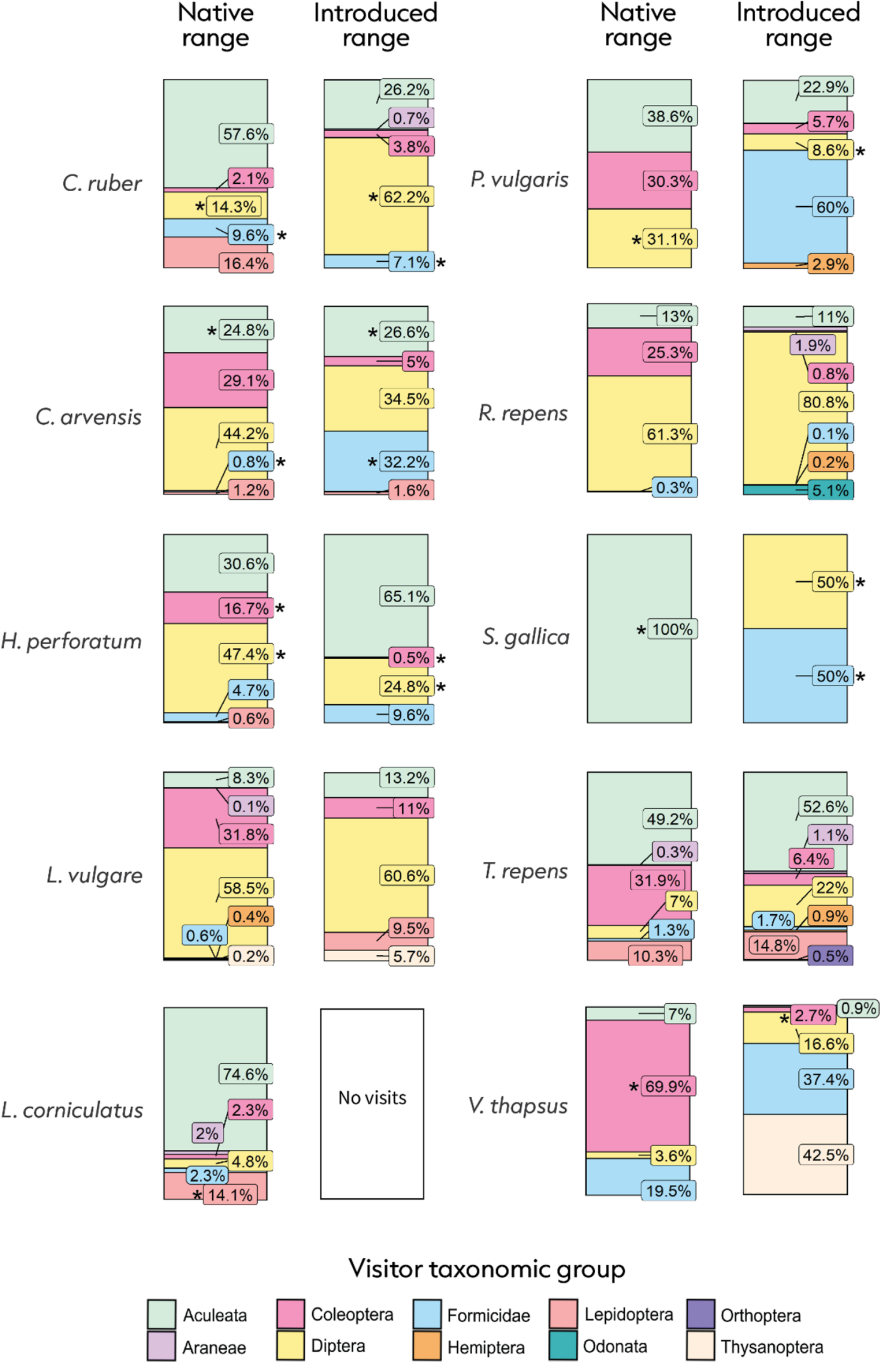
Comparison between ranges of the community composition of visitors per floral unit for each plant species. Significant differences (P < 0.05) are determined by multivariate abundance analyses and are denoted by asterisks (*) (values listed in Supplementary information).

In a study spanning four countries, two continents, and ten plant species, we observed 2652 flower visits (Fig. 2). We show that plants interact with 2.6 times more flower visitors in the native range than in the introduced range (estimate = 0.948, SE = 0.343, P = 0.006; Fig. 3a). Plants also interacted with a 1.8 times greater richness of flower visitors in the native range than in the introduced range (estimate = 0.569, SE = 0.22, P = 0.01; Fig. 3b). One species, *Lotus corniculatus*, had no observed visitors in the introduced range despite being visited by six different taxa in the native range. Seven of ten species showed evidence for flower visitor assemblage dissimilarity between their native and introduced ranges (P < 0.05; Fig. 4).

Our study provides the most comprehensive test of the Missed Mutualist Hypothesis to date^9,10,12,22–25^. The observed reduction in visitor abundance and richness might be substantial enough to impact plants’ reproductive potential in their introduced ranges^26^. However, the actual effect of missed mutualists could surpass what our study implies since we only sampled species that had managed to establish populations in a new range. Species reliant on pollinators that are absent in the introduced range would be incapable of establishing viable populations in their new habitat and, therefore, be excluded from our study.

A loss of flower visitors could help to explain the extremely high proportion of unsuccessful plant invasions^10^, as only ~ 25% of plant species successfully take consecutive steps in the invasion process (i.e., introduction, establishment, and spread)^27^. Introduced plants could overcome this disadvantage through evolution of a greater capacity for selfing^28–30^. For example, *Arctotheca populifolia*, a beach daisy native to South Africa, adapted to reproduce asexually in less than 200 years since its introduction to Australia and interaction with fewer pollinators^31^. A global study by Razanajatovo et al.^32^ further supports this idea, finding that selfing plants are more likely to establish in new ranges. Similarly, Pyšek et al.^1^ found that in Central Europe, selfing was the best reproductive strategy to facilitate invasive plants. Plants missing their mutualist visitors could evolve traits that increase visitation by new taxa^10^. Consistent with this idea, seven of our ten study species showed evidence for visitor assemblage dissimilarity between their native and introduced ranges (P < 0.05). Understanding visitor assemblages between ranges could give important new insights into the factors shaping the reproductive success and spread of introduced plants.

Visitation of introduced plants may not have decreased enough for the plants to be pollen limited, which could explain their ability to thrive despite missing mutualists. There is mixed evidence in the literature for an effect of visitation frequency and pollinator richness on the amount of seed set by plants^15,33–39^. While our plants may be encountering fewer, less diverse, and different pollinators, they could still be setting similar quantities of seeds if the pollinators that do interact with them are highly efficient and transfer high pollen loads (i.e., they are not pollen limited). There is evidence to suggest that some mutualistic interactions may even be enhanced in the introduced range and promote invasion, however, these studies only consider belowground mutualisms and it is currently unknown whether this concept translates across pollination^40,41^. Our study can prompt future work directed at examining pollen loads/deposition and fruit/seed production across ranges to disentangle specific drivers underpinning the success of introduced plant species.

A loss of flower visitors could have evolutionary consequences, possibly even the evolution of increased competitive ability (EICA)^10^. EICA is most commonly associated with enemy release, whereby introduced species gain a competitive edge over native species by reallocating energy from defence to growth and reproduction (Fig. 1), as a result of reduced herbivore pressure^4^. However, a reduction in mutualists, or certain types of mutualists, may also have a similar effect. For example, energy allocated to attracting specialised pollinators in the native range (i.e., costly nectar production, production of showy flowers, or long flower tubes), may be redirected into setting seeds of higher quality or quantity in the introduced range, an advantage not possessed by native competitors.

Our study focuses on entomophilous species whose flowers are visited by an array of generalist pollinators. However, not all introduced plant species adopt this reproductive strategy^42^. Asexually reproducing species (e.g., through rhizomes or stolons) alongside non-outcrossing or self-pollinating plants, do not rely on mutualistic floral interactions to ensure successful reproduction^43–46^. Coevolved interactions between flowers and visitors may also be highly specialised with successful pollinators constrained to taxa with certain morphologies (e.g., long proboscis to reach down nectar tubes) or belonging to specific clades^47,48^. More research is needed to understand if and how the missed mutualist hypothesis may apply across these different reproductive modes and their subsequent impact on invasion success.

### Introduced species lose more foe than friends

**Figure 5:**
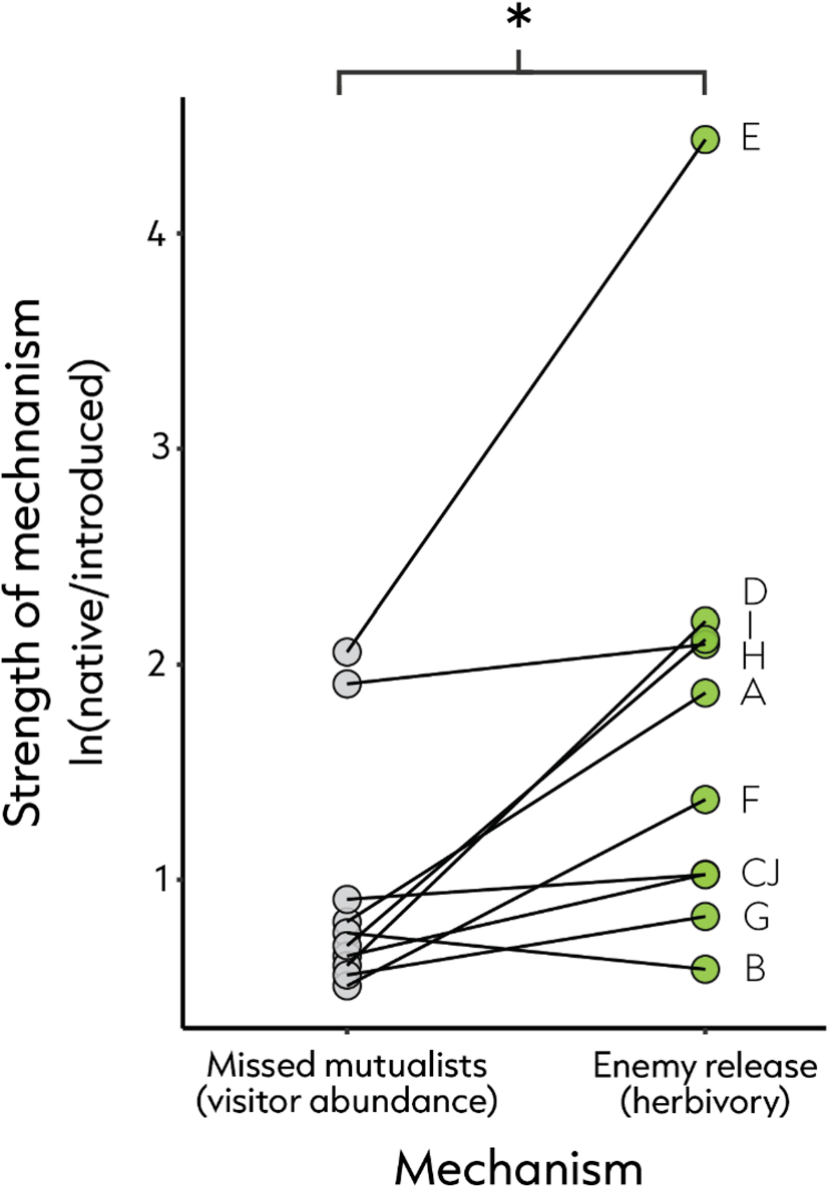
Paired comparison between the magnitude of effects for missed mutualists (grey) and enemy release (green) across 16 plant species (Paired T-test mean difference = 0.81, t = 3.19, *df* = 9, P = 0.01). Letters A-J correspond to plant species listed in Fig. 3.

A parallel study found that plant species experienced an average of 5.8 times more herbivory in their native range than in their introduced range^49^, while the present work revealed only 2.6 times more visitors in their native range (Paired T test mean difference = 0.81, t = 3.19, *df* = 9, P = 0.01; Fig. 5). That is, introduced plants seem to shed more enemies than mutualists. This may be because pollination is a mutually beneficial interaction posing little risk to naïve mutualists in the introduced range^36^. Conversely, generalist enemies may be more hesitant to ingest leaf material from unfamiliar non-native plants, as they pose a risk of harbouring deadly defences that can reduce herbivore fitness, or even result in herbivore mortality^50,51^. Another possibility is that the selective pressure on species to find alternative pathways for reproduction is more direct than the selective pressure resulting from herbivory. The fact that the benefits of enemy release outweigh the cost of missed mutualisms (Fig. 5) might help to explain the success of introduced plants.

Ecologists have long focused their attention on understanding how enemy release can facilitate successful invasion. Here, we show evidence that missed mutualisms are also likely to be important in the invasion process. Our findings present a valuable opportunity for ecologists to further explore whether other types of mutualisms are missed in the introduced range (e.g., plants and beneficial fungi or protective mites), and whether the interplay of both enemy release and missed mutualisms could more accurately predict which species, in what locations, would be more likely to invade natural ecosystems.

## Methods

### Data collection

To determine whether plants are interacting with fewer, less diverse pollinators in their introduced range we chose target species that were:

1. Biotically pollinated.
2. Present across a wide area in their native (Europe) and introduced ranges (Australia).

Using these criteria, we identified 15 plant species. Some species, however, were unable to be located or observed at least once in both ranges despite our best efforts in the field. Our final dataset is comprised of observations from the ten herbaceous plant species belonging to nine families and eight orders for which we were able to take observations in at least one site across both native and introduced ranges (Supplementary information).

**Figure 6:**
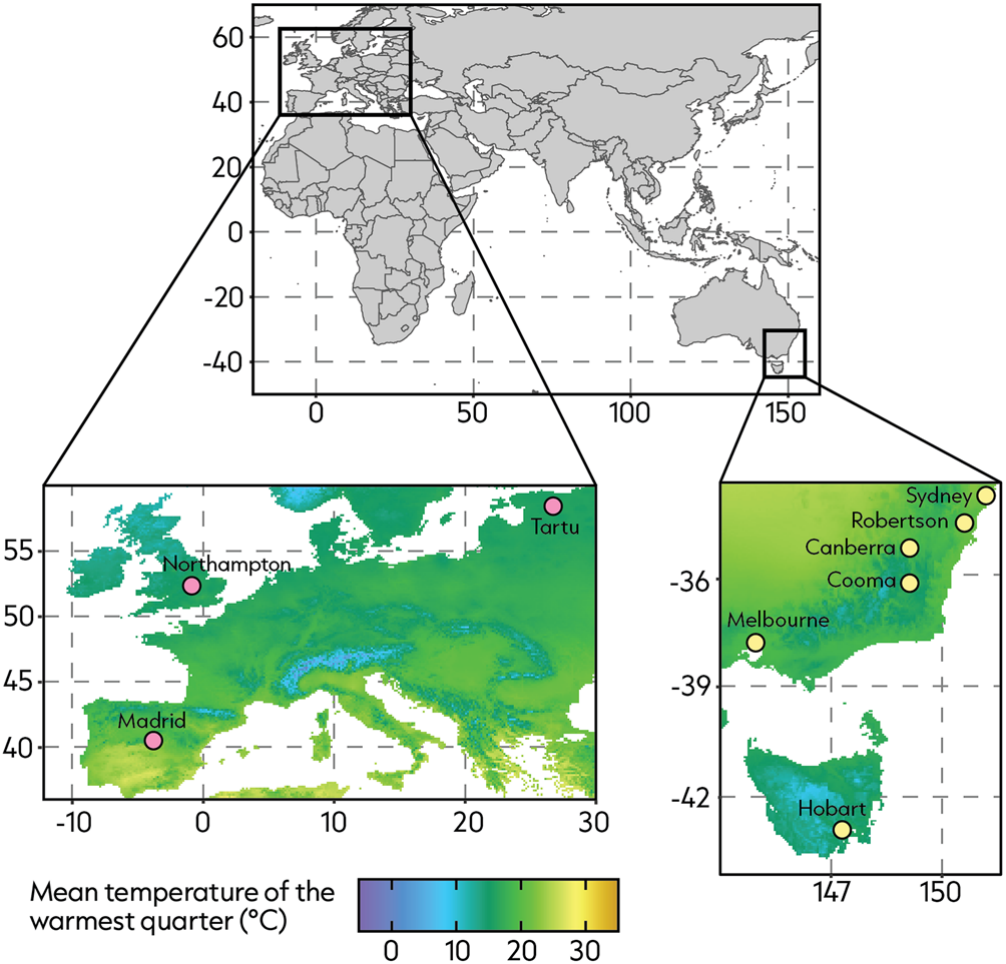
Maps of Europe (native range) and Australia (introduced range) where floral observations took place. [Bottom left] Sites in Europe include Madrid (Spain), Northampton (United Kingdom) and Tartu (Estonia). [Bottom right] Sites in Australia include Hobart (Tasmania), Melbourne (Victoria), Cooma (New South Wales), Canberra (Australian Capital Territory), Robertson (New South Wales), and Sydney (New South Wales). Maps are shaded according to mean temperature of the warmest quarter from WorldClim version 2.1 climate data for 1970–2000^64^, as it is the time of year when most pollination occurs^65^.

We conducted floral observations at nine sites within the native and introduced ranges of ten plant species. For most of our study species in Australia, the exact source population, or populations for multiple introductions (as in *Hypericum perforatum*^52^ and *Trifolium repens*^53^), are not known. Further, most of the introduced species present in Australia have very wide home ranges^54–63^. Thus, studying a single native population and comparing it with a single introduced population could give misleading results. We therefore selected a broad range of sites and climatic conditions to get a general understanding of plant-pollinator interactions within and across native and introduced ranges (Fig. 6). We prioritised maximising the latitudinal range and landscape diversity in each range when selecting our study sites. We also considered the presence of our target species when choosing sites as we favoured places that would increase our sampling potential. Prior to choosing site locations we used online resources such as the Global Biodiversity Information Facility (gbif.org) and the Atlas of Living Australia (ala.org.au) to check the presence of our target species. However, not all study species were present at each site (i.e., city or region where sampling took place) (see Supplementary information).

Floral observations were made in early to mid-summer of 2019 from May to August in Europe and from September to December in Australia. When choosing individuals, we looked for those that were actively in flower and then randomly selected individual plants from that subset population. This was done using a compass and random number generator to determine the observer’s direction of movement, the first individual encountered (or nearby) when walking in this direction was sampled. If ten or fewer individuals were present, all were sampled. All observations were conducted in daylight hours between 10am and 3 pm on days with no rain and minimal wind. Each observation was timed at 15 min and conducted with the observer placed 1–2 m from the target plant. We aimed to repeat these timed observations for at least 10 individuals of each species at every site. In total, we conducted 250 timed observations in the native range and 236 in the introduced range.

To be considered a pollinator, an animal must act as a vector for transferring pollen from one flower to another^66^. As we could not visually ensure successful pollination occurring during our observations, we instead quantified the number of times a flower was visited by invertebrates (potential pollinators). We defined a ‘visit’ as any time an invertebrate came into contact with floral reproductive organs (e.g., anthers or stigma), as implemented across similar field studies^67,68^. The duration of a visit was not recorded, and longer visits were considered the same as shorter ones (e.g., if a bee interacted with a flower for 5 min or 5 s it was still considered one visit). Visitors were categorised according to their taxonomic group as bees/wasps (Aculeata), flies (Diptera), ants (Formicidae), beetles (Coleoptera), butterflies/moths (Lepidoptera), spiders (Araneae), true bugs (Hemiptera), thrips (Thysanoptera), dragonflies (Odonata), and grasshoppers (Orthoptera). The total number of “floral units” (defined for each species in the Supplementary information) where visitors were observed on the target individual were also counted. If an invertebrate visited the same floral unit more than once (i.e., the animal left and then returned) or visited multiple floral units on the same individual, they were counted as multiple visits. Visitor abundance was calculated as the total number of visits from each taxonomic group for each observation. Visitor richness was defined as the number of taxonomic groups that were recorded for each observation.

Estimates of enemy release for our comparative analysis were sourced from Xirocostas et al.^49^.

### Data analysis

All statistical analyses were performed in RStudio version 4.2.0^69^. We included all groups of arthropods observed on floral units in our analyses, including those not (currently) known to be pollinators such as Odonata and Araneae^66^, because although they may not act as pollinators in Europe, it is not known if they play this role in Australia.

To test the hypothesis that plants in their introduced range will receive fewer visits to their flowers than conspecifics in the native range, we performed an overall comparison of visitor abundance between ranges for each species with Generalized Linear Mixed Models using Template Model Builder^70^. Our response variable was visitor abundance, and the predictor variable was range. We also included random effects terms for site and species. We offset our model by the log-transformed number of floral units per observation to account for this variance in affecting our abundance counts and used a negative binomial family as our data were over-dispersed.

To determine whether visitor richness was higher in plants’ native range than in the introduced range, we also fit a Generalized Linear Mixed Model. Our response variable was visitor richness and predictor variable was range; we also included random effects terms for site, species, and the polynomial-transformed number of floral units. We offset our model by the log-transformed number of floral units to account for this variance in affecting our richness counts and used a Poisson distribution as our data were not as over dispersed as the abundance data.

To determine whether the visitor assemblage of flower visitors differs between ranges we performed separate comparisons for each species across their native and introduced range. For each target species we created a community matrix of all observed visitor taxa using the *mvabund* function in the *mvabund* package^71^. Then we ran alternate and null hypothesis generalised linear models using the *manyglm* function. Our alternate models used the community matrix as the response variable, range as the predictor variable, and were offset by the log-transformed number of floral units to account for this variance across observations. Our null models were similar, except that our predictor variable (range) was removed and replaced with an intercept term (1). We then ran an ANOVA to calculate the distribution of our test statistic under the null hypothesis of no effect of range. Adjusted P values were calculated using 999 iterations via case block resampling with a stratified cluster bootstrap to account for correlation due to site.

To test whether plants experience differing degrees of enemy release in comparison to pollinator loss, we compared our floral visitation data with enemy release data from a parallel study^49^ conducted on the same plant species. For each species, herbivory data were collected by visually estimating leaf damage^72^ on a percentage scale of zero (no damage) to one hundred (complete consumption) on 10 leaves for at least 12 randomly selected individuals per site [full protocol in Xirocostas et al.^49^]. We then performed a paired samples t-test on the coefficients generated from the generalised linear mixed models performed in Xirocostas et al.^49^ and our study. These model coefficients represent the magnitude of the effect (log response ratio), for enemy release (herbivore damage) and missed mutualisms (flower visitor abundance) for each of our target species.

### Supplementary Information

Supplementary Information.

## Data Availability

Code and data associated with this study are available at the following links: https://doi.org/10.6084/m9.figshare.22819490.v1. https://doi.org/10.6084/m9.figshare.22819475.v1.

## References

[1] Pyšek P (2011). Successful invaders co-opt pollinators of native flora and accumulate insect pollinators with increasing residence time. Ecol. Monogr..

[2] Shine R (2010). The ecological impact of invasive Cane Toads (*Bufo marinus*) in Australia. Q. Rev. Biol..

[3] Vitousek PM, D’Antonio CM, Loope LL, Westbrooks R (1996). Biological invasions as global environmental change. Am. Sci..

[4] Blossey B, Nötzold R (1995). Evolution of increased competitive ability in invasive nonindigenous plants: A hypothesis. J. Ecol..

[5] Keane RM, Crawley MJ (2002). Exotic plant invasions and the enemy release hypothesis. Trends Ecol. Evol..

[6] Colautti RI, Ricciardi A, Grigorovich IA, MacIsaac HJ (2004). Is invasion success explained by the enemy release hypothesis?. Ecol. Lett..

[7] Crawley, M. J. What makes a community invasible? In *Colonization, succession and stability* (eds Gray A. J., Crawley M. J., Edwards P. J.) 429-453. Oxford, UK: Blackwell Scientific Publications (1987).

[8] Callaway RM, Ridenour WM (2004). Novel weapons: Invasive success and the evolution of increased competitive ability. Front. Ecol. Environ..

[9] Mitchell CE (2006). Biotic interactions and plant invasions. Ecol. Lett..

[10] Moles AT, Dalrymple RL, Raghu S, Bonser SP, Ollerton J (2022). Advancing the missed mutualist hypothesis, the under-appreciated twin of the enemy release hypothesis. Biol. Lett..

[11] Richardson DM, Allsopp N, D’antonio CM, Milton SJ, Rejmánek M (2000). Plant invasions—The role of mutualisms. Biol. Rev..

[12] Alpert P (2006). The advantages and disadvantages of being introduced. Biol. Invasions.

[13] Ollerton J, Winfree R, Tarrant S (2011). How many flowering plants are pollinated by animals?. Oikos.

[14] Ratto F (2018). Global importance of vertebrate pollinators for plant reproductive success: A meta-analysis. Front. Ecol. Environ..

[15] Albrecht M, Schmid B, Hautier Y, Müller CB (2012). Diverse pollinator communities enhance plant reproductive success. Proc. R. Soc. B Biol. Sci..

[16] Memmott J, Waser NM (2002). Integration of alien plants into a native flower–pollinator visitation web. Proc. R. Soc. Lond. Ser. B Biol. Sci..

[17] Vilà M (2009). Invasive plant integration into native plant–pollinator networks across Europe. Proc. R. Soc. B Biol. Sci..

[18] Morales CL, Aizen MA (2006). Invasive mutualisms and the structure of plant–pollinator interactions in the temperate forests of north-west Patagonia, Argentina. J. Ecol..

[19] Kaiser-Bunbury CN, Valentin T, Mougal J, Matatiken D, Ghazoul J (2011). The tolerance of island plant–pollinator networks to alien plants. J. Ecol..

[20] Olesen JM, Eskildsen LI, Venkatasamy S (2002). Invasion of pollination networks on oceanic islands: Importance of invader complexes and endemic super generalists. Divers. Distrib..

[21] Heger T, Jeschke JM (2014). The enemy release hypothesis as a hierarchy of hypotheses. Oikos.

[22] Dickie IA (2017). The emerging science of linked plant–fungal invasions. New Phytologist.

[23] Nuñez MA, Horton TR, Simberloff D (2009). Lack of belowground mutualisms hinders Pinaceae invasions. Ecology.

[24] Traveset, A. & Richardson, D. M. Mutualistic interactions and biological invasions. (2014).

[25] Zenni RD, Nuñez MA (2013). The elephant in the room: The role of failed invasions in understanding invasion biology. Oikos.

[26] Williamson M, Fitter A (1996). The varying success of invaders. Ecology.

[27] Jeschke JM, Pyšek P (2018). Tens rule. Invasion Biol. Hypotheses Evid..

[28] Kalisz S, Vogler DW (2003). Benefits of autonomous selfing under unpredictable pollinator environments. Ecology.

[29] Ollerton J (2012). Pollination ecology of the invasive tree tobacco *Nicotiana glauca*: Comparisons across native and non-native ranges. J. Pollinat. Ecol..

[30] Tabassum S, Leishman MR (2019). It doesn’t take two to tango: Increased capacity for self-fertilization towards range edges of two coastal invasive plant species in eastern Australia. Biol. Invasions.

[31] Brandenburger, C. R. Rapid evolution of an introduced plant. *PhD, UNSW Sydney* (2019).

[32] Razanajatovo M (2016). Plants capable of selfing are more likely to become naturalized. Nat. Commun..

[33] Albrecht M, Duelli P, Müller C, Kleijn D, Schmid B (2007). The Swiss agri-environment scheme enhances pollinator diversity and plant reproductive success in nearby intensively managed farmland. J. Appl. Ecol..

[34] Cohen H, Philpott SM, Liere H, Lin BB, Jha S (2021). The relationship between pollinator community and pollination services is mediated by floral abundance in urban landscapes. Urban Ecosyst..

[35] Gómez JM, Bosch J, Perfectti F, Fernández J, Abdelaziz M (2007). Pollinator diversity affects plant reproduction and recruitment: The tradeoffs of generalization. Oecologia.

[36] Harmon JP, Ganguli AC, Solga MJ (2011). An overview of pollination in rangelands: Who, why, and how. Rala.

[37] Hegland SJ, Totland Ø (2008). Is the magnitude of pollen limitation in a plant community affected by pollinator visitation and plant species specialisation levels?. Oikos.

[38] Klein A, Steffan-Dewenter I, Tscharntke T (2003). Fruit set of highland coffee increases with the diversity of pollinating bees. Proc. R. Soc. Lond. Ser. B Biol. Sci..

[39] Magrach A, González-Varo JP, Boiffier M, Vilà M, Bartomeus I (2017). Honeybee spillover reshuffles pollinator diets and affects plant reproductive success. Nat. Ecol. Evol..

[40] Reinhart KO, Callaway RM (2004). Soil biota facilitate exotic acer invasions in Europe and North America. Ecol. Appl..

[41] Reinhart KO, Callaway RM (2006). Soil biota and invasive plants. New Phytologist.

[42] Barrett SCH, Colautti RI, Eckert CG (2008). Plant reproductive systems and evolution during biological invasion. Mol. Ecol..

[43] He L (2021). Clonal fragments of stoloniferous invasive plants benefit more from stolon storage than their congeneric native species. Flora.

[44] French K (2021). Invasion by hawkweeds. Biol. Invasions.

[45] Maurer DA, Zedler JB (2002). Differential invasion of a wetland grass explained by tests of nutrients and light availability on establishment and clonal growth. Oecologia.

[46] Van Kleunen M, Johnson SD (2007). Effects of self-compatibility on the distribution range of invasive European plants in North America. Conserv. Biol..

[47] Arditti J, Elliott J, Kitching IJ, Wasserthal LT (2012). ‘Good Heavens what insect can suck it’—Charles Darwin, *Angraecum sesquipedale* and *Xanthopan morganii praedicta*. Bot. J. Linn. Soc..

[48] Johnson SD, Steiner KE (2000). Generalization versus specialization in plant pollination systems. Trends Ecol. Evol..

[49] Xirocostas ZA (2023). The great escape: Patterns of enemy release are not explained by time, space or climate. Proc. R. Soc. B Biol. Sci..

[50] Agrawal AA, Fishbein M (2006). Plant defense syndromes. Ecology.

[51] Moles AT (2011). Putting plant resistance traits on the map: A test of the idea that plants are better defended at lower latitudes. New Phytologist.

[52] Harris J, Gill A (1997). History of the introduction and spread of St. John’s wort (*Hypericum perforatum* L.) in Australia. Plant Prot. Q..

[53] Lane LA, Ayres JF, Lovett JV (1997). A review of the introduction and use of white clover (*Trifolium repens* L.) in Australia—Significance for breeding objectives. Aust. J. Exp. Agric..

[54] Austin DF (2000). Bindweed (*Convolvulus arvensis*, Convolvulaceae) in North America, from Medicine to Menace. J. Torrey Bot. Soc..

[55] Dieskau J, Bruelheide H, Gutknecht J, Erfmeier A (2020). Biogeographic differences in plant–soil biota relationships contribute to the exotic range expansion of *Verbascum thapsus*. Ecol. Evol..

[56] Geerts S, Rossenrode T, Irlich UM, Visser V (2017). Emerging ornamental plant invaders in urban areas—Centranthus ruber in Cape Town, South Africa as a case study. Invasive Plant Sci. Manag..

[57] Kooyers NJ, Olsen KM (2013). Searching for the bull’s eye: Agents and targets of selection vary among geographically disparate cyanogenesis clines in white clover (*Trifolium repens* L.). Heredity.

[58] Kozminska A (2018). Comparative analysis of water deficit and salt tolerance mechanisms in Silene. S. Afr. J. Bot..

[59] Mimura M, Ono K, Goka K, Hara T (2013). Standing variation boosted by multiple sources of introduction contributes to the success of the introduced species, *Lotus corniculatus*. Biol. Invasions.

[60] Qu L, Widrlechner MP (2011). Variation in the breeding system of *Prunella vulgaris* L. HortScience.

[61] Stutz S, Mráz P, Hinz HL, Müller-Schärer H, Schaffner U (2018). Biological invasion of oxeye daisy (*Leucanthemum vulgare*) in North America: Pre-adaptation, post-introduction evolution, or both?. PLoS ONE.

[62] Vilà M, Maron JL, Marco L (2005). Evidence for the enemy release hypothesis in *Hypericum perforatum*. Oecologia.

[63] Warren J (2009). Extra petals in the buttercup (*Ranunculus repens*) provide a quick method to estimate the age of meadows. Ann. Bot..

[64] Fick SE, Hijmans RJ (2017). WorldClim 2: New 1-km spatial resolution climate surfaces for global land areas. Int. J. Climatol..

[65] Tooke F, Battey NH (2010). Temperate flowering phenology. J. Exp. Bot..

[66] Ollerton J (2021). Pollinators and Pollination: Nature and Society.

[67] Chrobock T (2013). Effects of native pollinator specialization, self-compatibility and flowering duration of European plant species on their invasiveness elsewhere. J. Ecol..

[68] Chrobock T, Winiger P, Fischer M, van Kleunen M (2013). The cobblers stick to their lasts: Pollinators prefer native over alien plant species in a multi-species experiment. Biol. Invasions.

[69] R Core Team. R: A language and environment for statistical computing. R Foundation for Statistical Computing (2021).

[70] Brooks ME (2017). glmmTMB balances speed and flexibility among packages for zero-inflated generalized linear mixed modeling. R J..

[71] Wang, Y., Naumann, U., Wright, S. T. & Warton, D. I. mvabund—an R package for model-based analysis of multivariate abundance data. *Methods Ecol. Evol.* **3**, 471-474 (2019).

[72] Xirocostas ZA, Debono SA, Slavich E, Moles AT (2022). The ZAX herbivory trainer—Free software for training researchers to visually estimate leaf damage. Methods Ecol. Evol..

