# Supplementary material for: Introduced species shed friends as well as enemies
Source: Sci Rep. 2024 May 15;14:11088. doi: 10.1038/s41598-024-61788-8 (PMC11096385; doi:10.1038/s41598-024-61788-8)
Supplement: Supplementary file 1 — Supplementary Information. [file 41598_2024_61788_MOESM1_ESM.docx]

**Supplementary Information**

**Introduced species lose more enemies than friends**

Zoe A. Xirocostas ^1*†^, Jeff Ollerton^2, 3^, Begoña Peco^4^, Eve Slavich^5^, Stephen P. Bonser^1^, Meelis Pärtel^6^, S. Raghu^7^, and Angela T. Moles^1^

^1^Evolution & Ecology Research Centre, School of Biological, Earth and Environmental Sciences, UNSW Sydney, NSW 2052, Australia

^2^Kunming Institute of Botany, Chinese Academy of Sciences, Kunming, China

^3^Faculty of Arts, Science and Technology, University of Northampton, Northampton, UK

^4^Terrestrial Ecology Group (TEG), Department of Ecology, Institute for Biodiversity and Global Change, Universidad Autónoma de Madrid, 28049, Madrid, Spain

^5^Stats Central, Mark Wainwright Analytical Centre, UNSW Sydney, NSW 2052, Australia

^6^Institute of Ecology and Earth Sciences, University of Tartu, J. Liivi 2, 50409, Tartu, Estonia

^7^CSIRO Health & Biosecurity, Brisbane, QLD, Australia

^*^Corresponding author – email: [zoe.xirocostas@uts.edu.au](mailto:zoe.xirocostas@uts.edu.au)

^†^Current address – School of Life Sciences, Faculty of Science, University of Technology Sydney, NSW 2007, Australia

**Supplementary Tables**

**Supplementary information table 1.** Target species and their observed occurrences at each introduced site.

|  | **Introduced Range** | | | | | |
| --- | --- | --- | --- | --- | --- | --- |
| Target species | Canberra | Cooma | Hobart | Melbourne | Robertson | Sydney |
| *Centranthus ruber* | 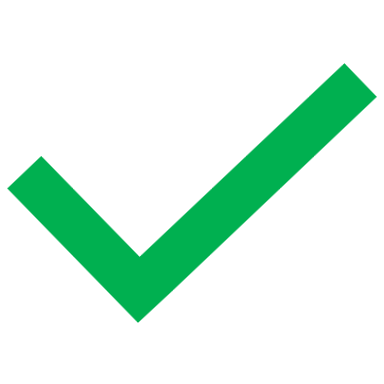 | 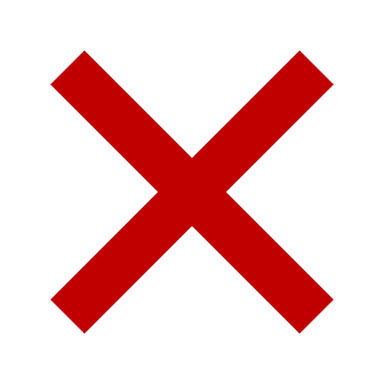 | 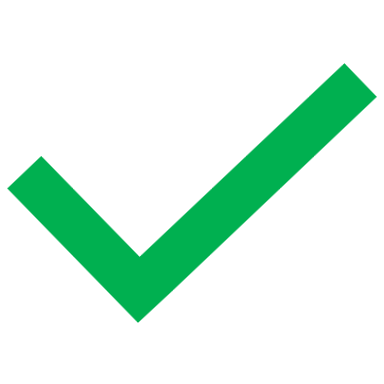 | 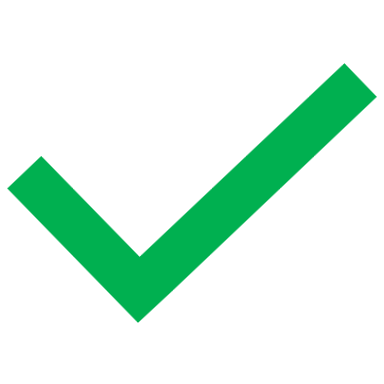 | 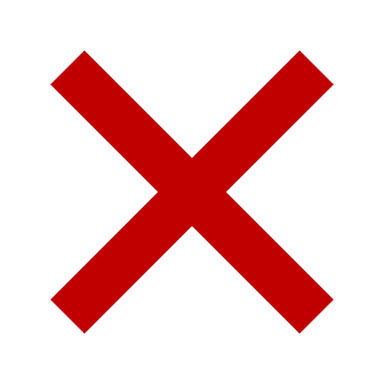 | 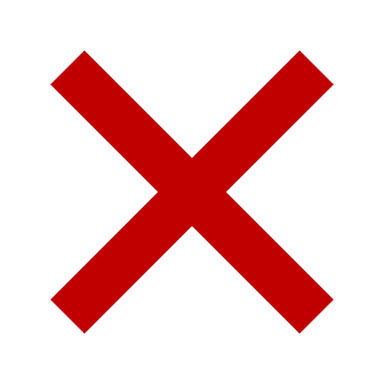 |
| *Convolvulus arvensis* | 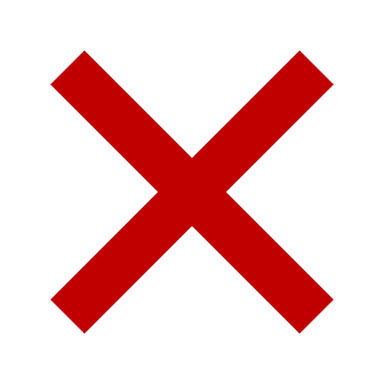 | 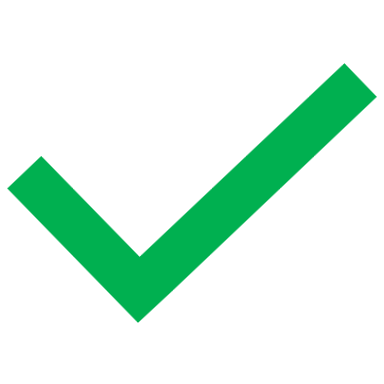 | 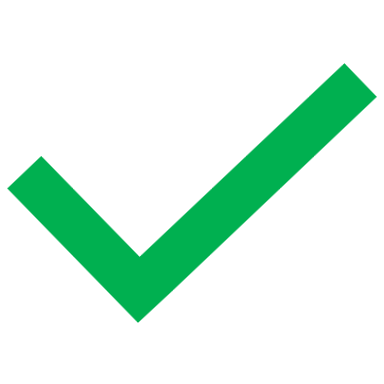 | 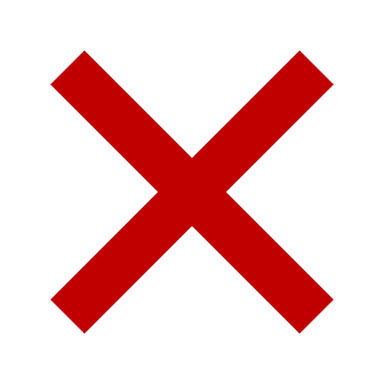 | 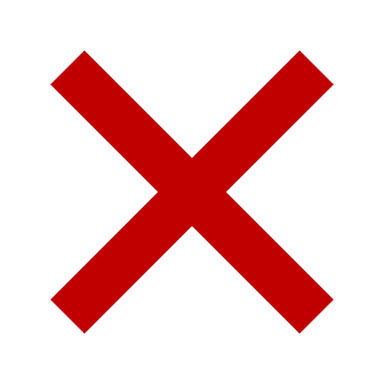 | 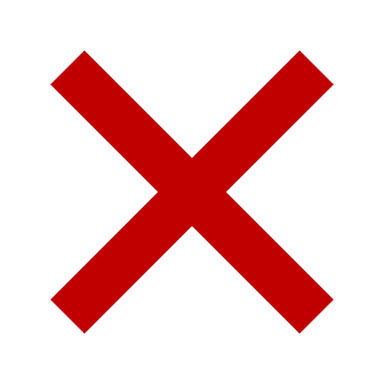 |
| *Hypericum perforatum* | 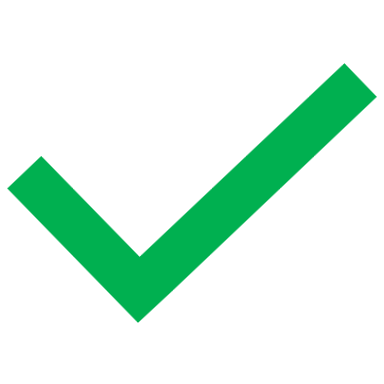 | 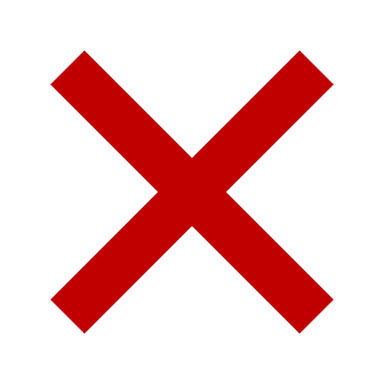 | 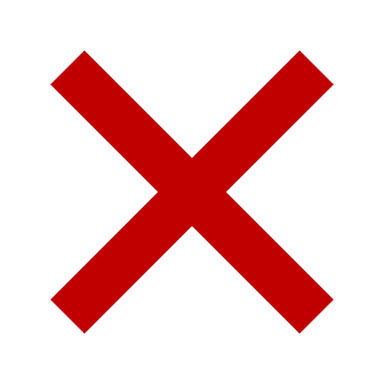 | 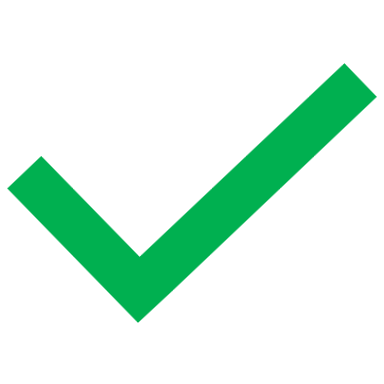 | 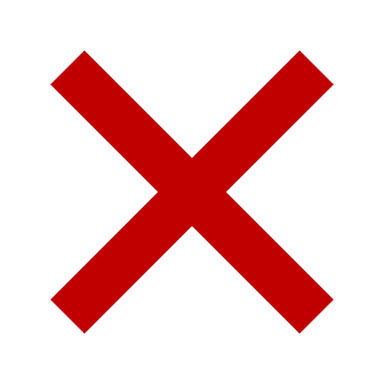 | 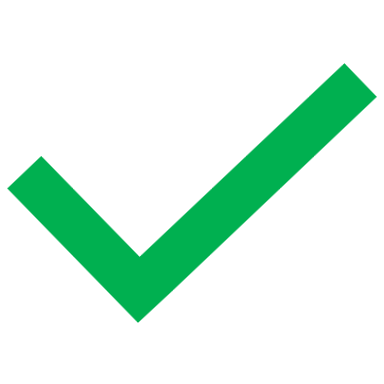 |
| *Leucanthemum vulgare* | 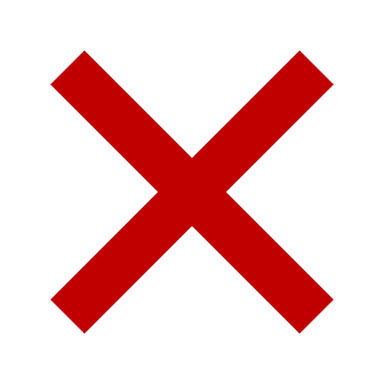 | 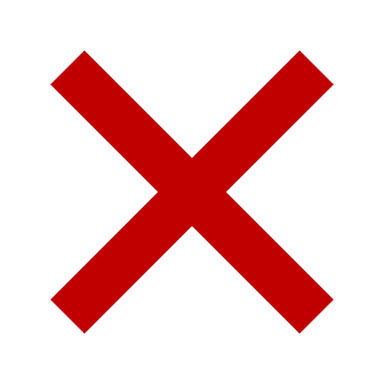 | 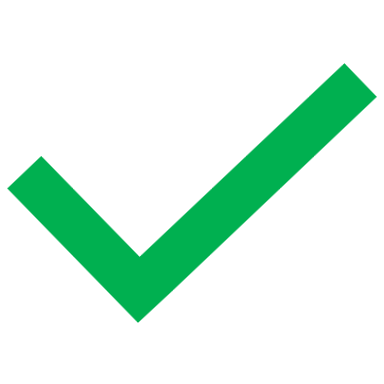 | 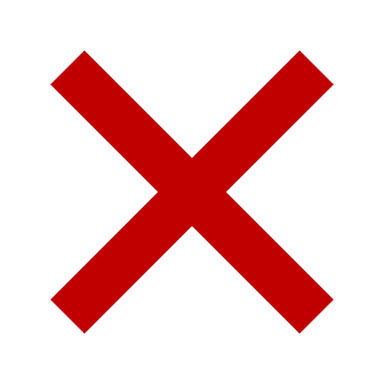 | 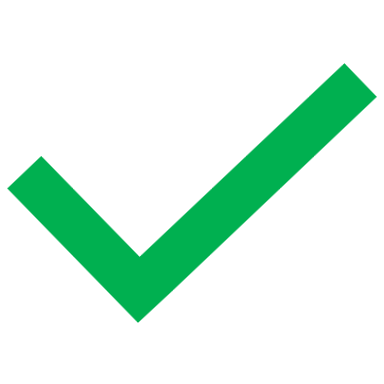 | 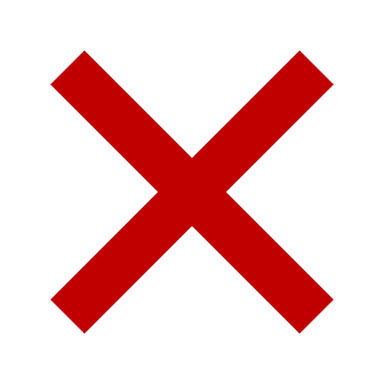 |
| *Lotus corniculatus* | 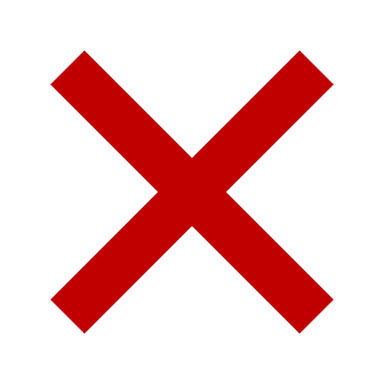 | 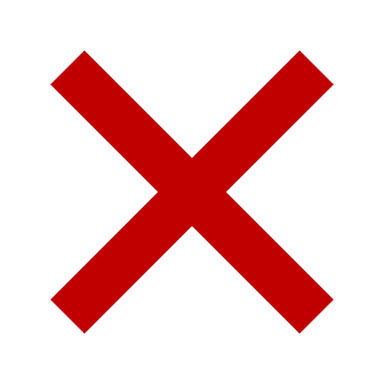 | 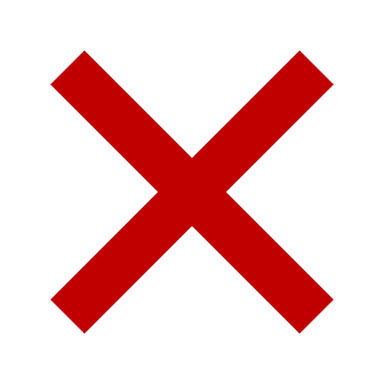 | 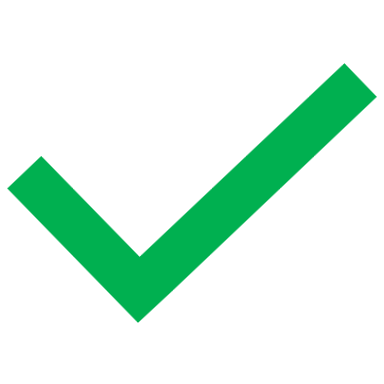 | 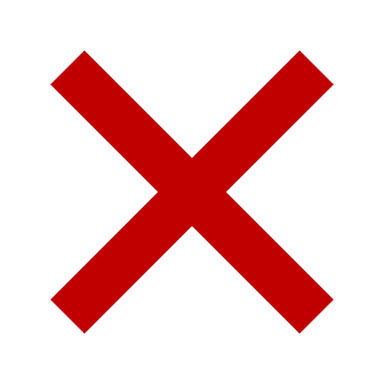 | 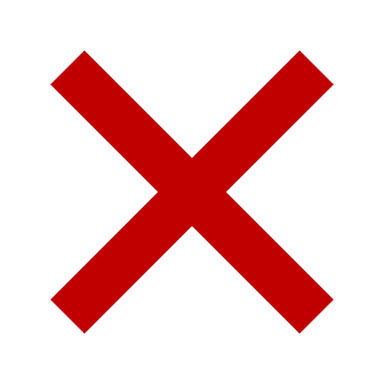 |
| *Prunella vulgaris* | 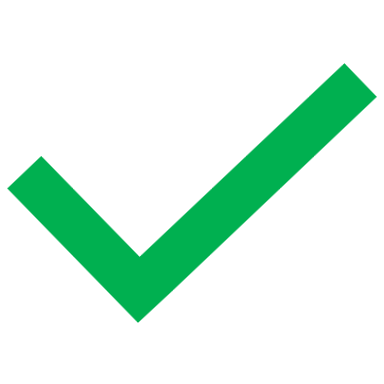 | 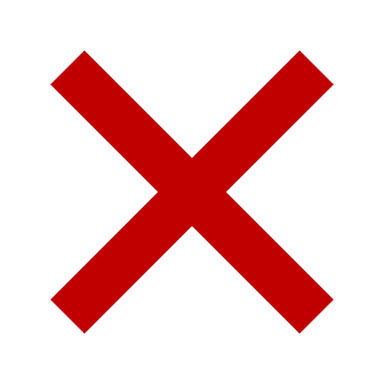 | 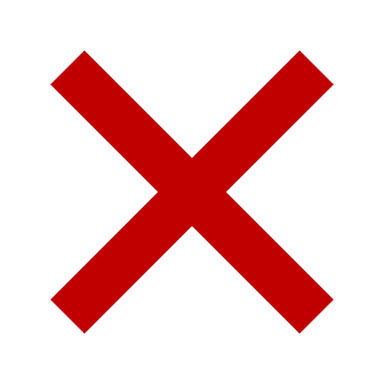 | 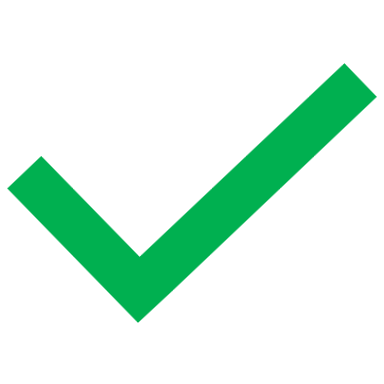 | 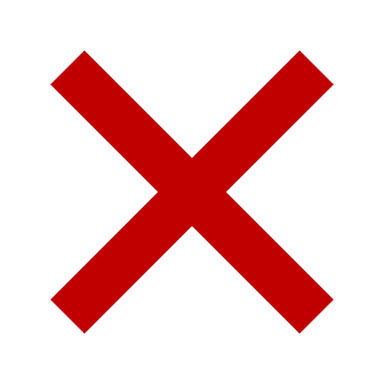 | 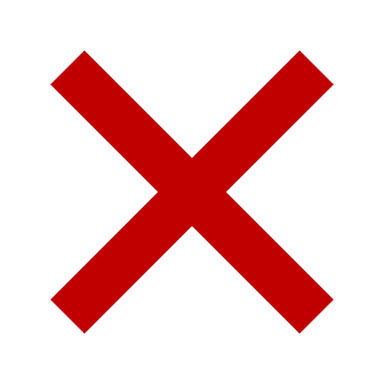 |
| *Ranunculus repens* | 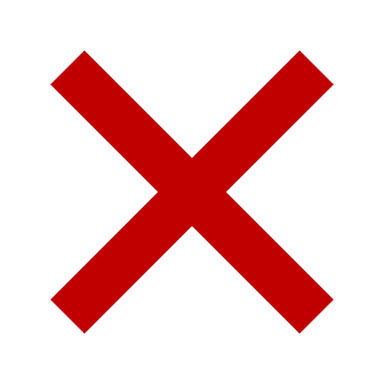 | 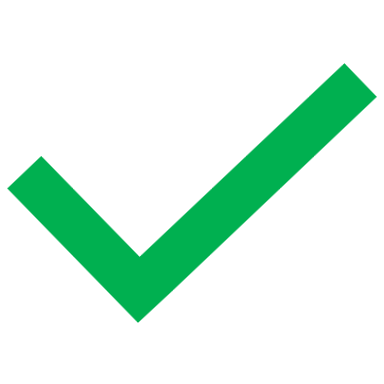 | 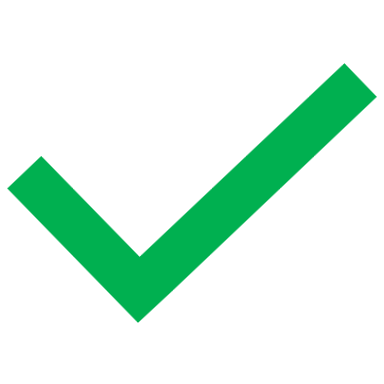 | 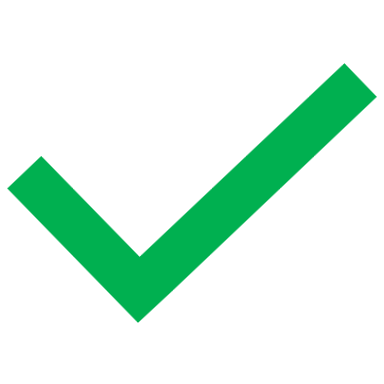 | 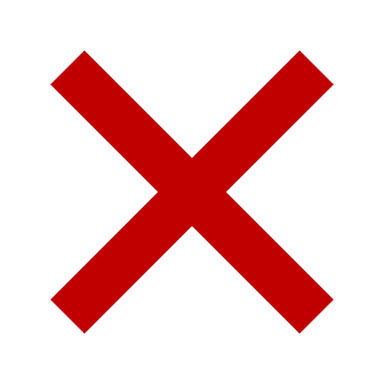 | 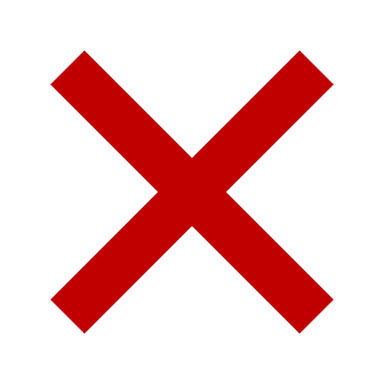 |
| *Silene gallica* | 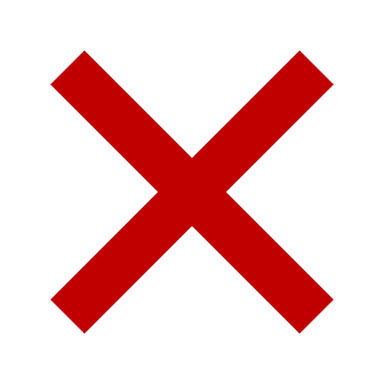 | 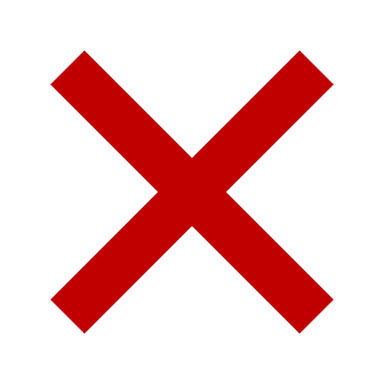 | 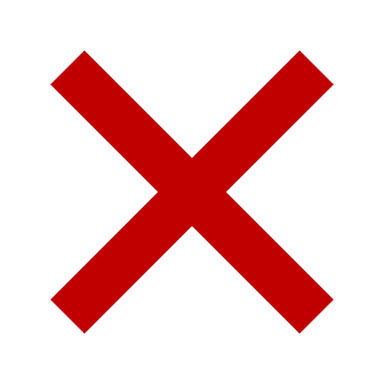 | 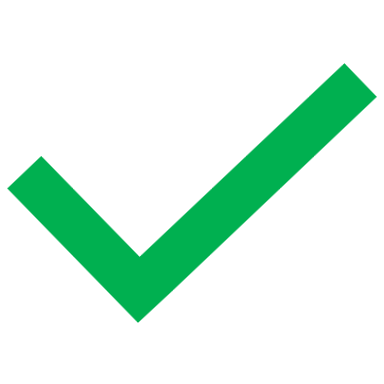 | 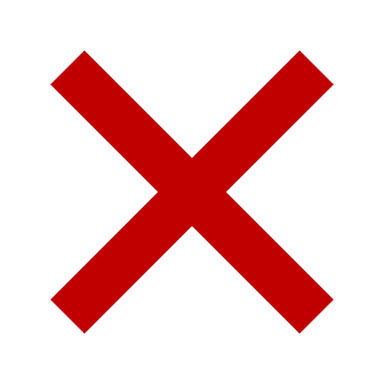 | 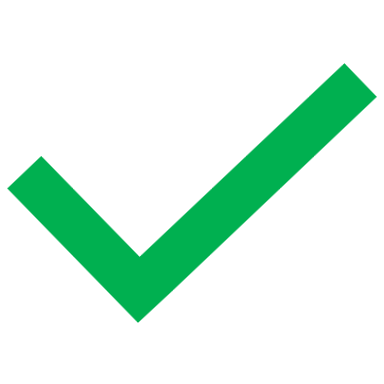 |
| *Trifolium repens* | 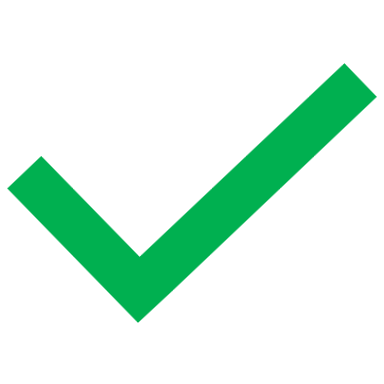 | 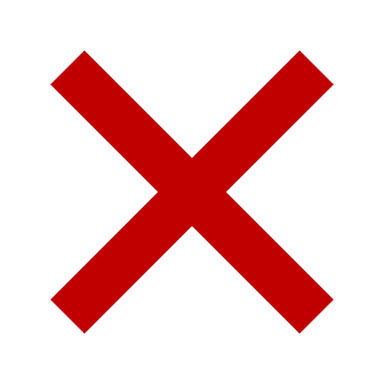 | 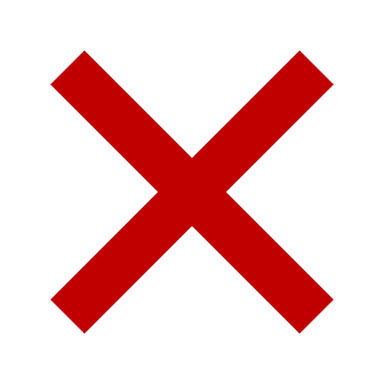 | 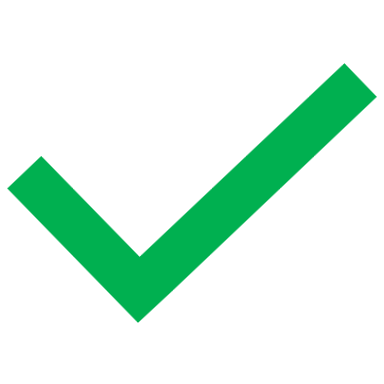 | 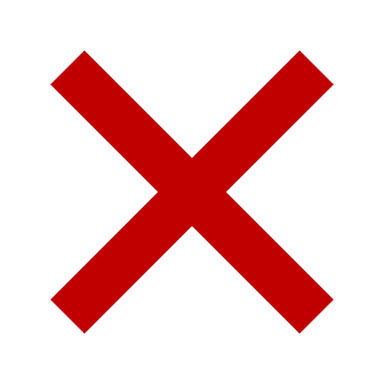 | 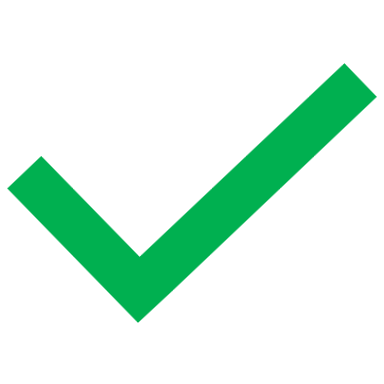 |
| *Verbascum thapsus* | 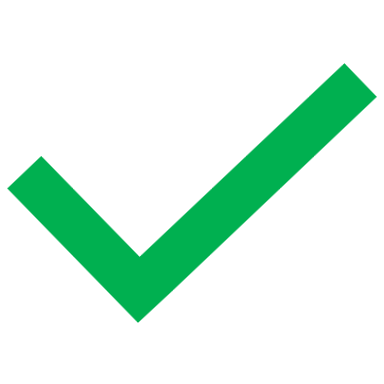 | 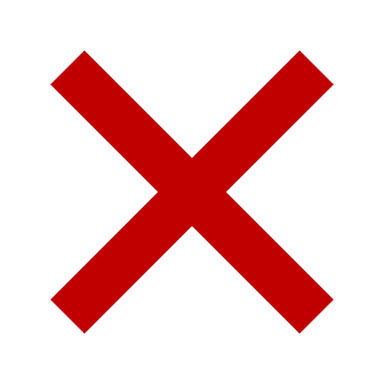 | 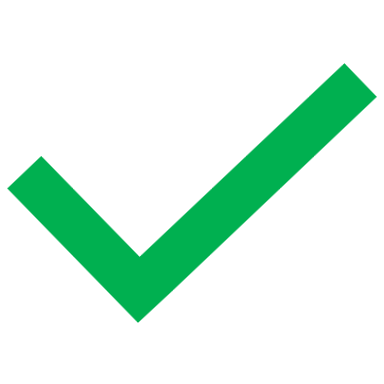 | 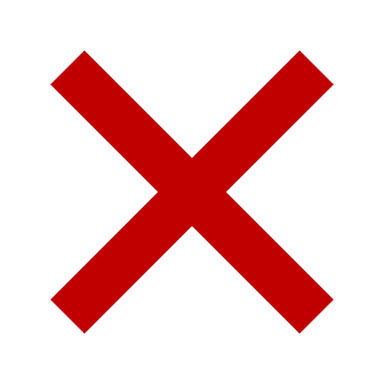 | 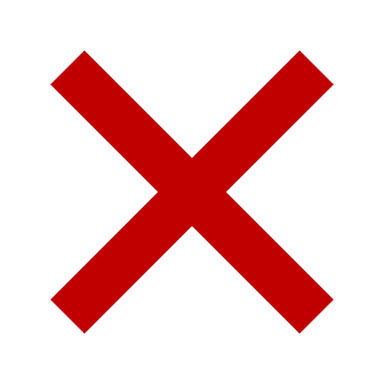 | 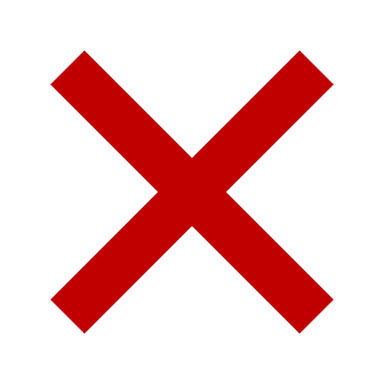 |

**Supplementary information table 2.** Target species and their observed occurrences at each native site.

|  | **Native Range** | | |
| --- | --- | --- | --- |
| Target species | Madrid | Northampton | Tartu |
| *Centranthus ruber* | 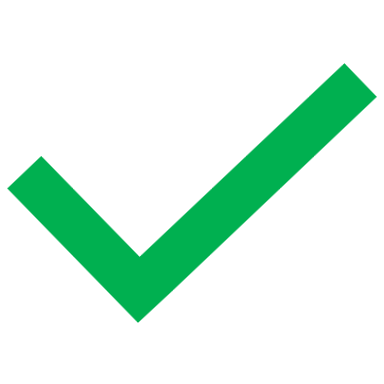 | 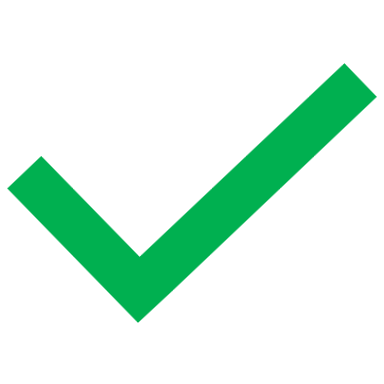* | 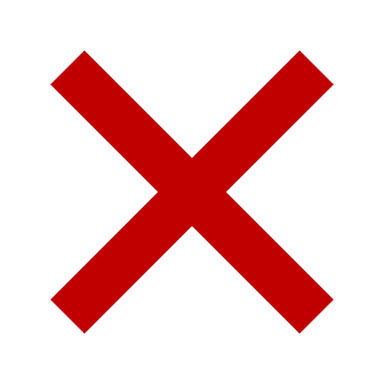 |
| *Convolvulus arvensis* | 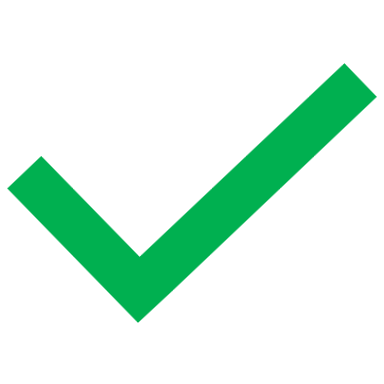 | 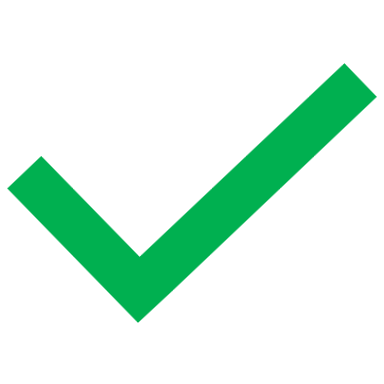 | 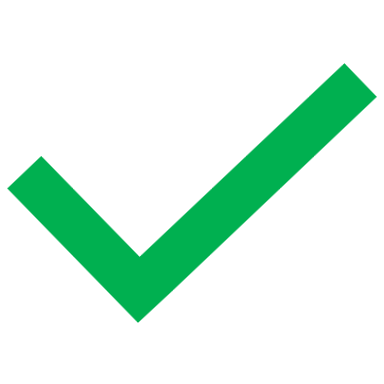 |
| *Hypericum perforatum* | 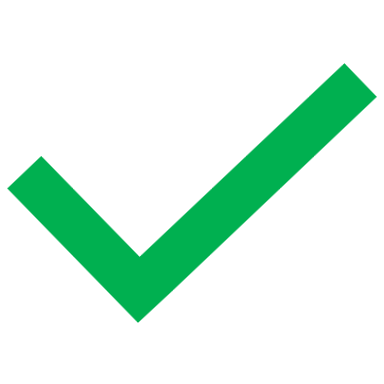 | 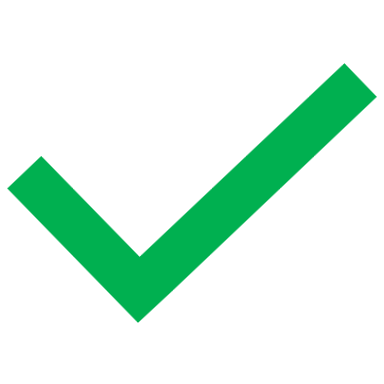 | 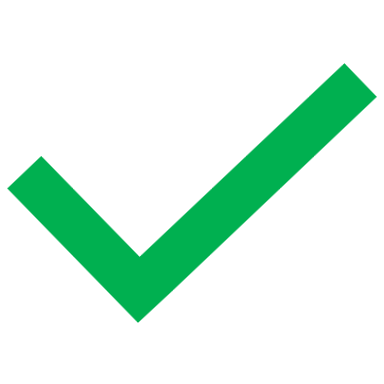 |
| *Leucanthemum vulgare* | 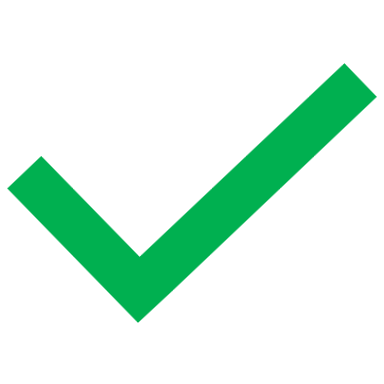 | 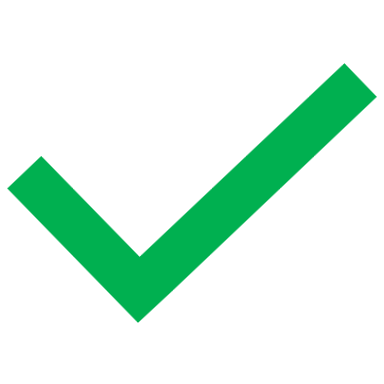 | 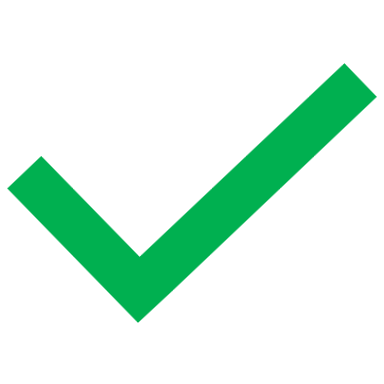 |
| *Lotus corniculatus* | 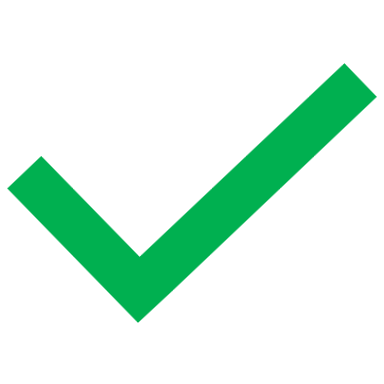 | 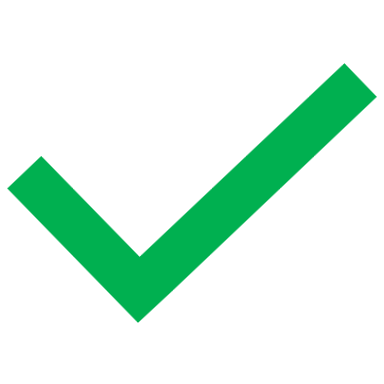 | 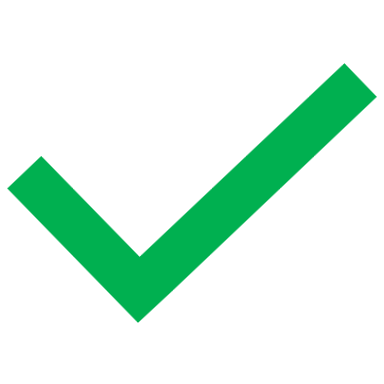 |
| *Prunella vulgaris* | 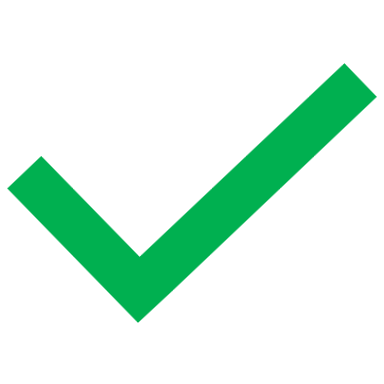 | 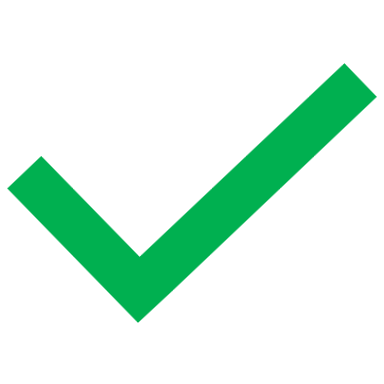 | 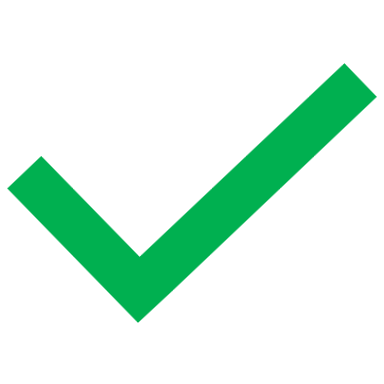 |
| *Ranunculus repens* | 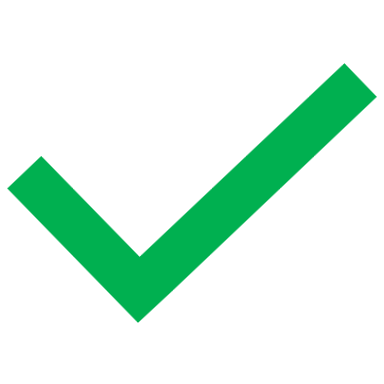 | 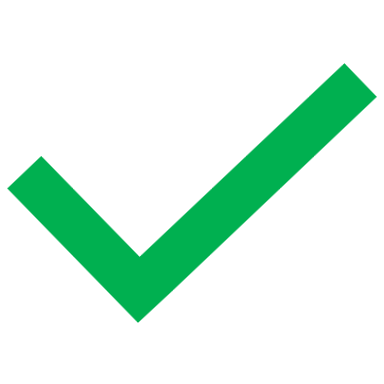 | 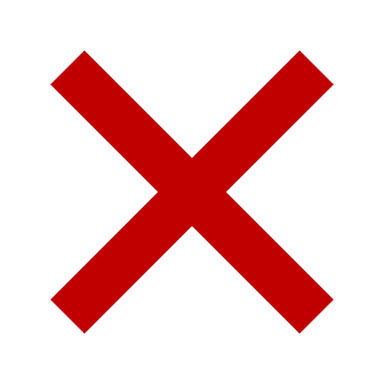 |
| *Silene gallica* | 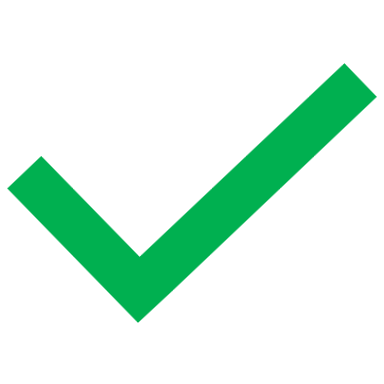 | 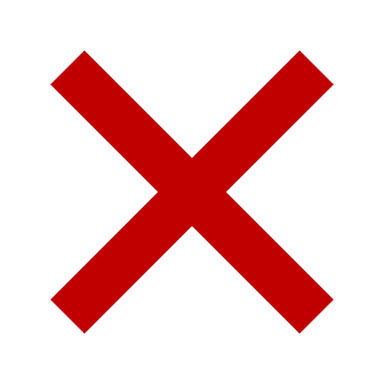 | 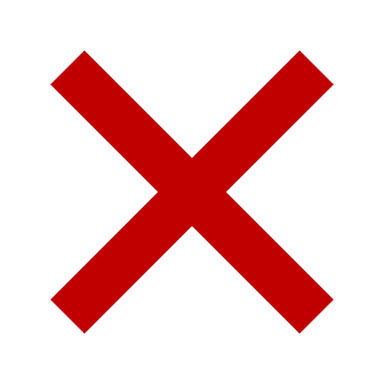 |
| *Trifolium repens* | 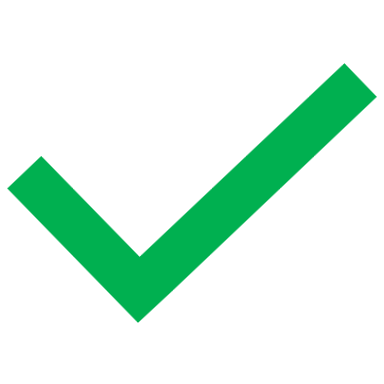 | 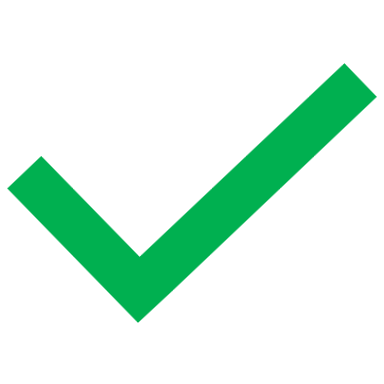 | 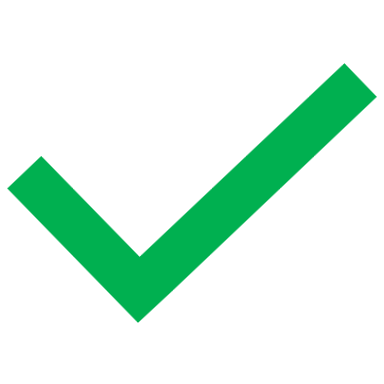 |
| *Verbascum thapsus* | 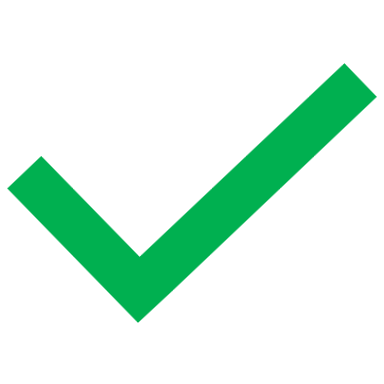 | 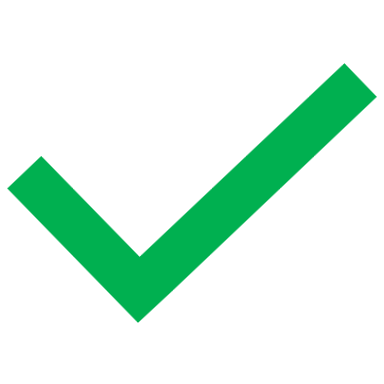 | 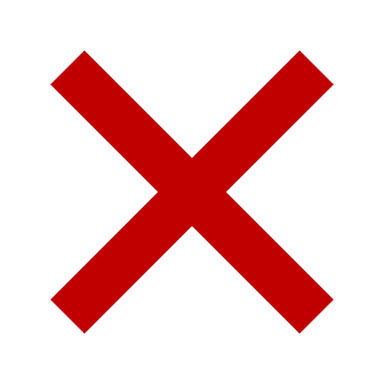 |

* We excluded pollination observations for *Centranthus ruber* in Northampton from our dataset as the close proximity (~900 km) to its native geographical origins of southern Europe, coupled with its early introduction to the UK (first recorded in 1597) caused ambiguity as to whether Northampton should be considered the native or introduced range for this species (Dwyer, 2021; Holmes et al., 2018). When excluding these points, we also considered that Australian introduced sites were ~15 times further from *C. ruber*’s native range of Europe (>14000 km) and that they were introduced over 200 years later than they had been in Northampton (Dwyer, 2021). References are listed at the end of the Supplementary Information.

**Supplementary information table 3.** Location specifics for sampling sites.

| **Site** | **Species** | **Location** | **Latitude** | **Longitude** |
| --- | --- | --- | --- | --- |
| Canberra | *Centranthus ruber* | Australian National University, Sullivans Creek Road, Acton, ACT 2601, Australia | -35.2795 | 149.1153 |
| Canberra | *Hypericum perforatum* | Australian National University, Acton, ACT 2601, Australia | -35.2813 | 149.1104 |
| Canberra | *Trifolium repens* | Black Mountain Peninsula, Clunies Ross Street Exit, Acton, ACT 2601, Australia | -35.2841 | 149.1094 |
| Canberra | *Prunella vulgaris* | Australian National University, Daley Road, Acton, ACT 2601, Australia | -35.2813 | 149.1126 |
| Canberra | *Verbascum thapsus* | 37 Naas Road, Paddys River, ACT 2620, Australia | -35.5205 | 149.0699 |
| Cooma | *Convolvulus arvensis* | 41 Commissioner Street, Cooma, NSW 2630, Australia | -36.2341 | 149.1301 |
| Cooma | *Ranunculus repens* | 73 Sharp Street, Cooma, NSW 2630, Australia | -36.2336 | 149.1283 |
| Hobart | *Centranthus ruber* | Lower Domain Road, Queens Domain, TAS 7000, Australia | -42.8656 | 147.3281 |
| Hobart | *Convolvulus arvensis* | 15 Marieville Esplanade, Sandy Bay, TAS 7005, Australia | -42.8982 | 147.332 |
| Hobart | *Leucanthemum vulgare* | 82 Channel Highway, Taroona, TAS 7053, Australia | -42.9389 | 147.3531 |
| Hobart | *Ranunculus repens* | 24-26 Weld Street, South Hobart, TAS 7004, Australia | -42.8921 | 147.3103 |
| Hobart | *Verbascum thapsus* | Tasman Highway, Buckland, TAS 7190, Australia | -42.5764 | 147.8178 |
| Madrid | *Centranthus ruber* | Calle de la Cruz de los Caídos, 1, 28792 Miraflores de la Sierra, Madrid, Spain | 40.8137 | -3.7698 |
| Madrid | *Convolvulus arvensis* | M-610, 28720 Bustarviejo, Madrid, Spain | 40.8555 | -3.7311 |
| Madrid | *Hypericum perforatum* | 28794 Guadalix de la Sierra, Madrid, Spain | 40.7773 | -3.6681 |
| Madrid | *Leucanthemum vulgare* | Carretera de Miraflores de la Sierra a Bustarviejo, 28720 Bustarviejo, Madrid, Spain | 40.8282 | -3.7585 |
| Madrid | *Lotus corniculatus* | M-610, 28720 Bustarviejo, Madrid, Spain | 40.8563 | -3.7423 |
| Madrid | *Prunella vulgaris* | M-633, 28729 Valdemanco, Madrid, Spain | 40.8559 | -3.656 |
| Madrid | *Ranunculus repens* | 28720 Bustarviejo, Madrid, Spain | 40.856 | -3.7403 |
| Madrid | *Silene gallica* | M-610, 28720 Bustarviejo, Madrid, Spain | 40.8563 | -3.7423 |
| Madrid | *Trifolium repens* | M-610, 28720 Bustarviejo, Madrid, Spain | 40.8563 | -3.7423 |
| Madrid | *Verbascum thapsus* | 28751 La Cabrera, Madrid, Spain | 40.8659 | -3.6279 |
| Melbourne | *Centranthus ruber* | 21 Bayview Ave, Clayton, VIC 3168, Australia | -37.9069 | 145.126 |
| Melbourne | *Hypericum perforatum* | 836 Wellington Road, Lysterfield, VIC 3156, Australia | -37.9445 | 145.3221 |
| Melbourne | *Lotus corniculatus* | 55 Therese Avenue, Mount Waverley, VIC 3149, Australia | -37.8919 | 145.1203 |
| Melbourne | *Prunella vulgaris* | Damper Creek Reserve, Mount Waverley, VIC, Australia | -37.8638 | 145.1277 |
| Melbourne | *Ranunculus repens* | Fairway Reserve, Scotchmans Creek Trail, Mount Waverley, VIC 3149, Australia | -37.8903 | -37.8903 |
| Melbourne | *Silene gallica* | 840 Wellington Rd, Lysterfield, VIC 3156, Australia | -37.9433 | 145.322 |
| Melbourne | *Trifolium repens* | Damper Creek Reserve, Mount Waverley, VIC, Australia | -37.8638 | 145.1277 |
| Northampton* | *Centranthus ruber ** | The Manor House, Broad St, Great Cambourne, Cambourne, Cambridge CB23 6DH, UK | 52.2197 | -0.0715 |
| Northampton | *Convolvulus arvensis* | UoN Waterside Campus, UK | 52.2302 | -0.8867 |
| Northampton | *Hypericum perforatum* | UoN Waterside Campus, UK | 52.2302 | -0.8867 |
| Northampton | *Leucanthemum vulgare* | UoN Waterside Campus, UK | 52.2302 | -0.8867 |
| Northampton | *Lotus corniculatus* | UoN Waterside Campus, UK | 52.2302 | -0.8867 |
| Northampton | *Prunella vulgaris* | UoN Waterside Campus, UK | 52.2302 | -0.8867 |
| Northampton | *Ranunculus repens* | UoN Waterside Campus, UK | 52.2302 | -0.8867 |
| Northampton | *Trifolium repens* | UoN Waterside Campus, UK | 52.2302 | -0.8867 |
| Northampton | *Verbascum thapsus* | UoN Waterside Campus, UK | 52.2302 | -0.8867 |
| Robertson | *Leucanthemum vulgare* | 3 Alcorn Lane, Robertson, NSW 2577, Australia | -34.5893 | 150.5879 |
| Sydney | *Hypericum perforatum* | 182-188 Carmichael Drive, West Hoxton, NSW 2171, Australia | -33.9306 | 150.8369 |
| Sydney | *Silene gallica* | 24 Centaur Street, Revesby, NSW 2212, Australia | -33.958 | 151.0213 |
| Sydney | *Trifolium repens* | Virginius Reserve, Padstow, NSW 2211, Australia | -33.9586 | 151.0227 |
| Tartu | *Convolvulus arvensis* | 61212 Konguta, Estonia | 58.2588 | 26.3535 |
| Tartu | *Hypericum perforatum* | 61207 Konguta, Estonia | 58.2696 | 26.3914 |
| Tartu | *Leucanthemum vulgare* | 61215 Vahessaare, Estonia | 58.2872 | 26.3577 |
| Tartu | *Lotus corniculatus* | 61207 Konguta, Estonia | 58.2696 | 26.3914 |
| Tartu | *Prunella vulgaris* | 61212 Konguta, Estonia | 58.2588 | 26.3535 |
| Tartu | *Trifolium repens* | 61202 Annikoru, Estonia | 58.2769 | 26.3032 |

**Supplementary table 4.** P-values for multivariate abundance analyses comparing visitor assemblages in plants’ native and introduced ranges while accounting for variance due to site and offset by number of floral units. Significant values are denoted by asterisks (*) and are adjusted for multiple testing.

| **Target species** | **Visitor taxonomic group** | **Deviance** | **P-value** |
| --- | --- | --- | --- |
| *Centranthus ruber* | Aculeata | 1.27 | 0.143 |
| *Centranthus ruber* | Diptera | 23.126 | 0.001* |
| *Centranthus ruber* | Coleoptera | 0.946 | 0.143 |
| *Centranthus ruber* | Lepidoptera | 1.808 | 0.143 |
| *Centranthus ruber* | Formicidae | 2.385 | 0.04* |
| *Centranthus ruber* | Araneae | 0.425 | 0.143 |
| *Convolvulus arvensis* | Aculeata | 13.661 | 0.02* |
| *Convolvulus arvensis* | Diptera | 7.195 | 0.078 |
| *Convolvulus arvensis* | Coleoptera | 0.36 | 0.642 |
| *Convolvulus arvensis* | Lepidoptera | 2.379 | 0.186 |
| *Convolvulus arvensis* | Formicidae | 11.59 | 0.039* |
| *Hypericum perforatum* | Aculeata | 0.91 | 0.574 |
| *Hypericum perforatum* | Diptera | 15.31 | 0.012* |
| *Hypericum perforatum* | Coleoptera | 11.297 | 0.013* |
| *Hypericum perforatum* | Lepidoptera | 1.499 | 0.435 |
| *Hypericum perforatum* | Formicidae | 0.576 | 0.574 |
| *Leucanthemum vulgare* | Aculeata | 2.825 | 0.726 |
| *Leucanthemum vulgare* | Diptera | 0.252 | 0.746 |
| *Leucanthemum vulgare* | Coleoptera | 5.604 | 0.726 |
| *Leucanthemum vulgare* | Lepidoptera | 5.473 | 0.632 |
| *Leucanthemum vulgare* | Formicidae | 3.484 | 0.725 |
| *Leucanthemum vulgare* | Hemiptera | 2.427 | 0.726 |
| *Leucanthemum vulgare* | Araneae | 1.176 | 0.726 |
| *Leucanthemum vulgare* | Thysanoptera | 1.264 | 0.726 |
| *Lotus corniculatus* | Aculeata | 0.38 | 0.558 |
| *Lotus corniculatus* | Diptera | 0.038 | 0.558 |
| *Lotus corniculatus* | Coleoptera | 1.29 | 0.37 |
| *Lotus corniculatus* | Lepidoptera | 30.494 | 0.048* |
| *Lotus corniculatus* | Formicidae | 0.934 | 0.371 |
| *Lotus corniculatus* | Araneae | 0.354 | 0.558 |
| *Prunella vulgaris* | Aculeata | 3.153 | 0.228 |
| *Prunella vulgaris* | Diptera | 21.814 | 0.001* |
| *Prunella vulgaris* | Coleoptera | 3.507 | 0.228 |
| *Prunella vulgaris* | Formicidae | 0.899 | 0.228 |
| *Prunella vulgaris* | Hemiptera | 0.651 | 0.228 |
| *Ranunculus repens* | Aculeata | 7.069 | 0.322 |
| *Ranunculus repens* | Diptera | 0.505 | 0.829 |
| *Ranunculus repens* | Coleoptera | 17.199 | 0.096 |
| *Ranunculus repens* | Formicidae | 0.781 | 0.829 |
| *Ranunculus repens* | Hemiptera | 2.304 | 0.544 |
| *Ranunculus repens* | Araneae | 2.22 | 0.544 |
| *Ranunculus repens* | Odonata | 4.755 | 0.322 |
| *Silene gallica* | Aculeata | 0.344 | 0.001* |
| *Silene gallica* | Diptera | 0.387 | 0.001* |
| *Silene gallica* | Formicidae | 0.387 | 0.001* |
| *Trifolium repens* | Aculeata | 2.986 | 0.569 |
| *Trifolium repens* | Diptera | 4.767 | 0.477 |
| *Trifolium repens* | Coleoptera | 16.705 | 0.133 |
| *Trifolium repens* | Lepidoptera | 0.397 | 0.906 |
| *Trifolium repens* | Formicidae | 0.48 | 0.906 |
| *Trifolium repens* | Hemiptera | 5.349 | 0.451 |
| *Trifolium repens* | Araneae | 0.588 | 0.906 |
| *Trifolium repens* | Orthoptera | 1.587 | 0.798 |
| *Verbascum thapsus* | Aculeata | 3.635 | 0.121 |
| *Verbascum thapsus* | Diptera | 12.259 | 0.073 |
| *Verbascum thapsus* | Coleoptera | 21.397 | 0.016* |
| *Verbascum thapsus* | Formicidae | 2.294 | 0.122 |
| *Verbascum thapsus* | Thysanoptera | 1.845 | 0.122 |

**Supplementary table 5.** Confidence intervals and model coefficients for each species (and overall) within the visitor abundance model.

| **Target species** | **Model coefficient** | **Standard error of model coefficient** | **Upper confidence interval** | **Lower confidence interval** |
| --- | --- | --- | --- | --- |
| *Centranthus ruber* | 0.804688 | 0.810741 | 2.393741 | -0.78436 |
| *Convolvulus arvensis* | 0.753209 | 0.787794 | 2.297285 | -0.79087 |
| *Hypericum perforatum* | 0.646188 | 0.787333 | 2.189361 | -0.89698 |
| *Leucanthemum vulgare* | 0.60156 | 0.827003 | 2.222487 | -1.01937 |
| *Lotus corniculatus* | 2.057609 | 1.028052 | 4.072592 | 0.042626 |
| *Prunella vulgaris* | 0.507032 | 1.042019 | 2.549389 | -1.53533 |
| *Ranunculus repens* | 0.557431 | 1.029424 | 2.575103 | -1.46024 |
| *Silene gallica* | 1.909468 | 1.0511 | 3.969623 | -0.15069 |
| *Trifolium repens* | 0.693934 | 1.25371 | 3.151205 | -1.76334 |
| *Verbascum thapsus* | 0.908593 | 1.083966 | 3.033166 | -1.21598 |
| Overall effect | 0.947652 | 0.343073 | 1.620075 | 0.27523 |

**Supplementary table 6.** Confidence intervals and estimated marginal means for each species (and overall) within the visitor richness model.

| **Target species** | **Model coefficient** | **Standard error of model coefficient** | **Upper confidence interval** | **Lower confidence interval** |
| --- | --- | --- | --- | --- |
| *Centranthus ruber* | 0.580386 | 0.681765 | 1.916645 | -0.75587 |
| *Convolvulus arvensis* | 0.425985 | 0.60314 | 1.608139 | -0.75617 |
| *Hypericum perforatum* | 0.566169 | 0.601967 | 1.746023 | -0.61369 |
| *Leucanthemum vulgare* | 0.448399 | 0.640421 | 1.703625 | -0.80683 |
| *Lotus corniculatus* | 0.812843 | 0.745538 | 2.274097 | -0.64841 |
| *Prunella vulgaris* | 0.630191 | 0.762884 | 2.125444 | -0.86506 |
| *Ranunculus repens* | 0.422007 | 0.756207 | 1.904174 | -1.06016 |
| *Silene gallica* | 0.968999 | 0.762814 | 2.464114 | -0.52612 |
| *Trifolium repens* | 0.333938 | 0.872026 | 2.04311 | -1.37523 |
| *Verbascum thapsus* | 0.448891 | 0.789746 | 1.996793 | -1.09901 |
| Overall effect | 0.568962 | 0.219843 | 0.999855 | 0.138069 |

**Supplementary Methods**

***Centranthus ruber***

For this species we considered one inflorescence as a floral unit. If an invertebrate was to visit multiple flowers within the same floral unit it would count as one visit. If the animal visited several floral units, each would be recorded as a visit.


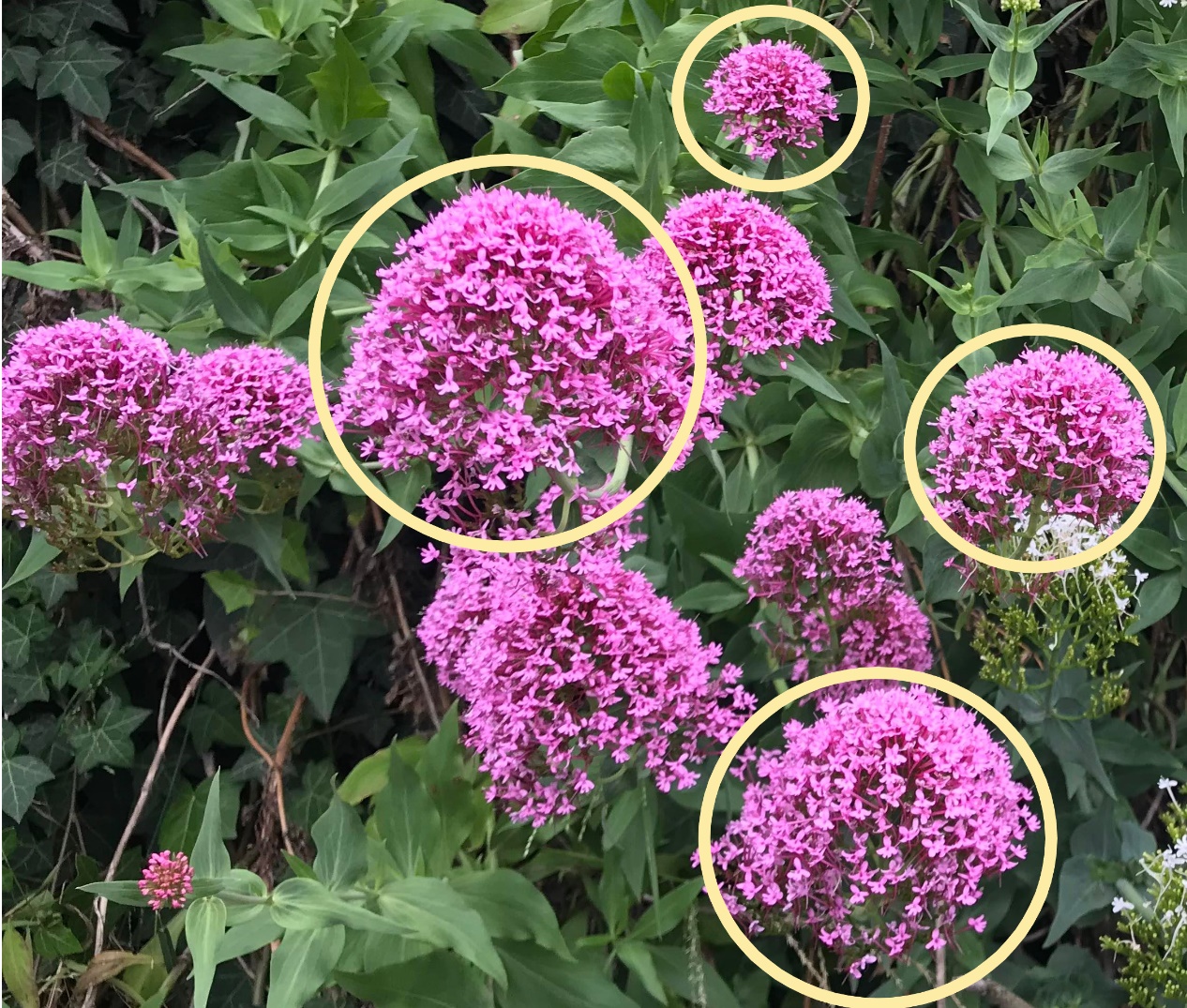


**Supplementary figure 1**. Image of *Centranthus ruber* in flower with floral units circled (Image credit: Zoe Xirocostas).

***Convolvulus arvensis***

For this species we considered one flower as a floral unit.


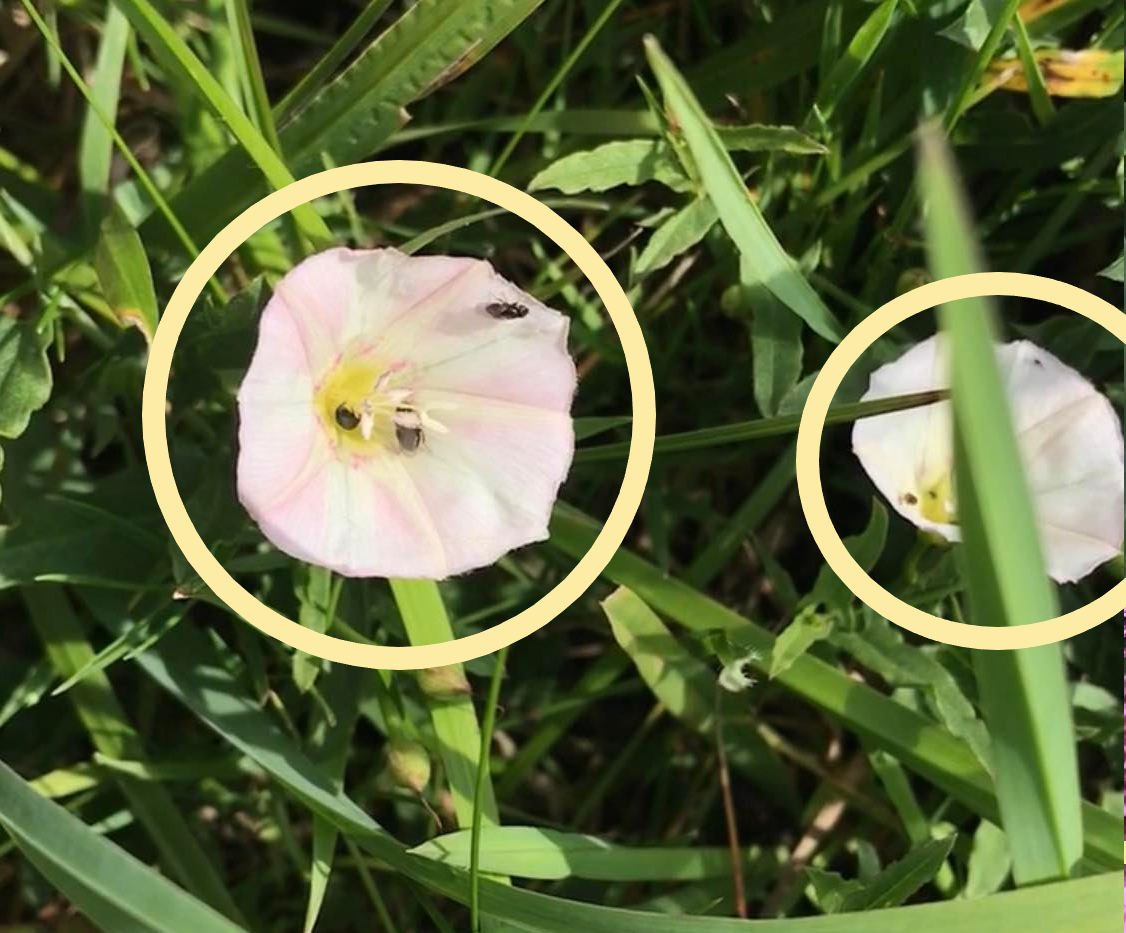


**Supplementary figure 2.** Image of *Convolvulus arvensis* in flower with floral units circled (Image credit: Zoe Xirocostas).

***Hypericum perforatum***

For this species we considered one flower as a floral unit.


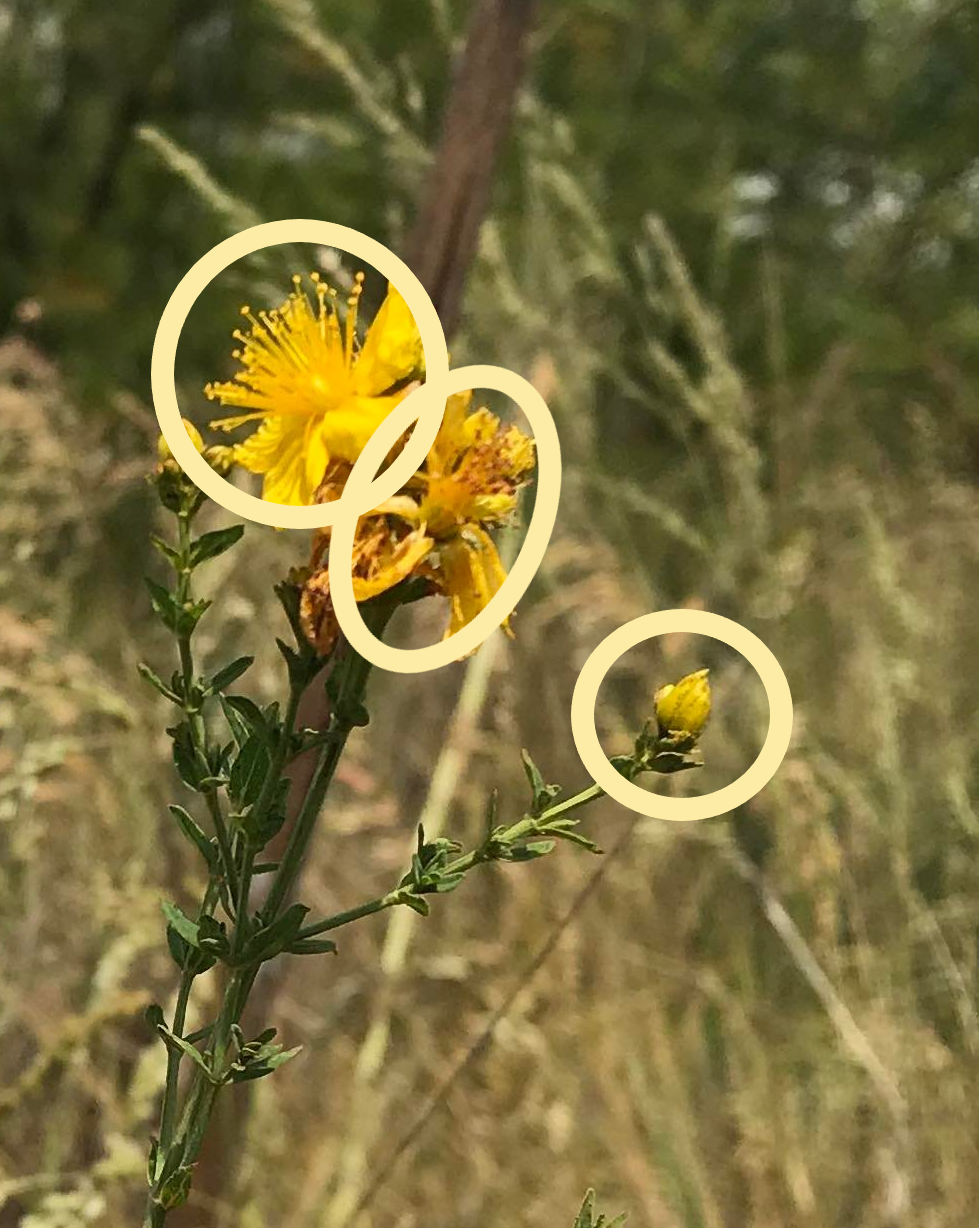


**Supplementary figure 3.** Image of *Hypericum perforatum* with floral units, in flower and emerging, circled (Image credit: Zoe Xirocostas).

***Leucanthemum vulgare***

For this species we considered one composite flower head as a floral unit.


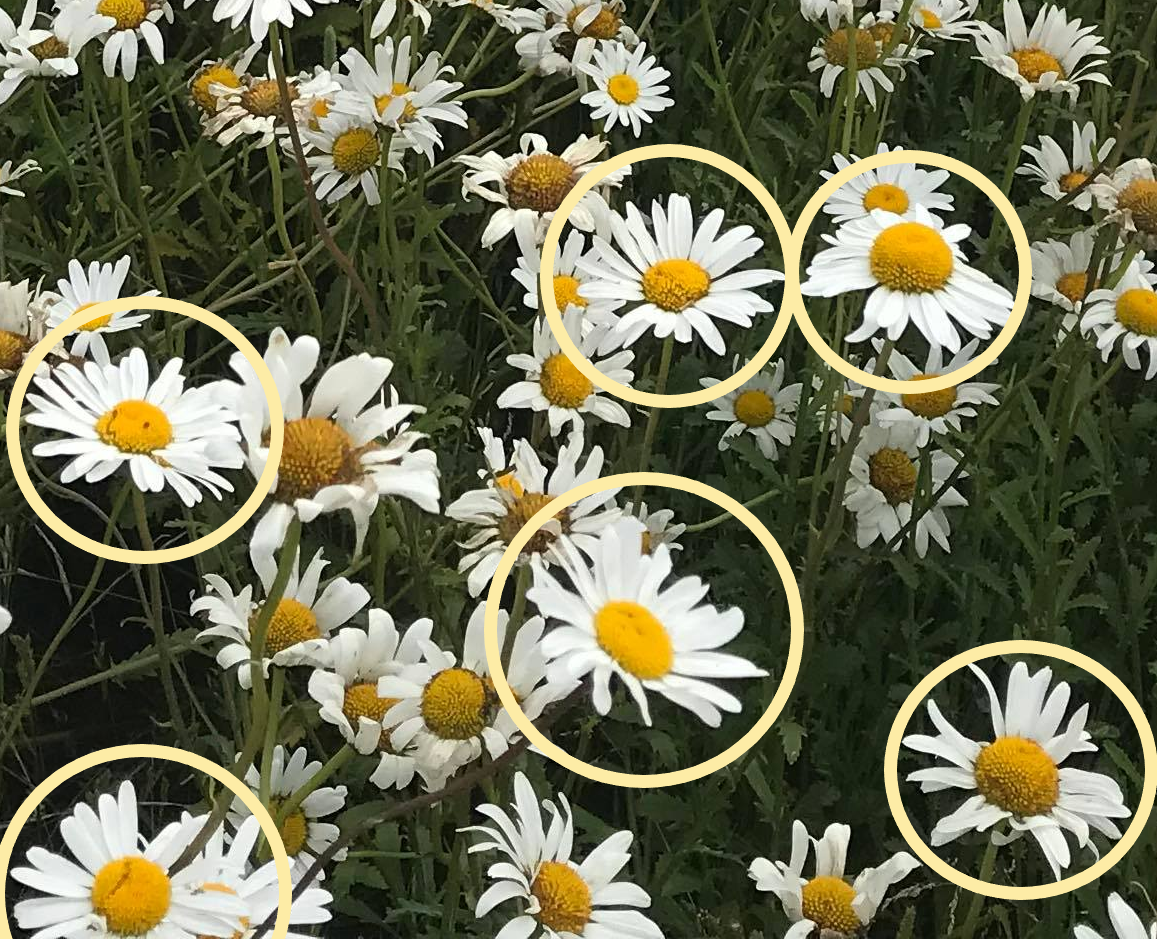


**Supplementary figure 4.** Image of *Leucanthemum vulgare* in flower with floral units circled (Image credit: Zoe Xirocostas).

***Lotus corniculatus***

For this species we considered each flower as a floral unit.


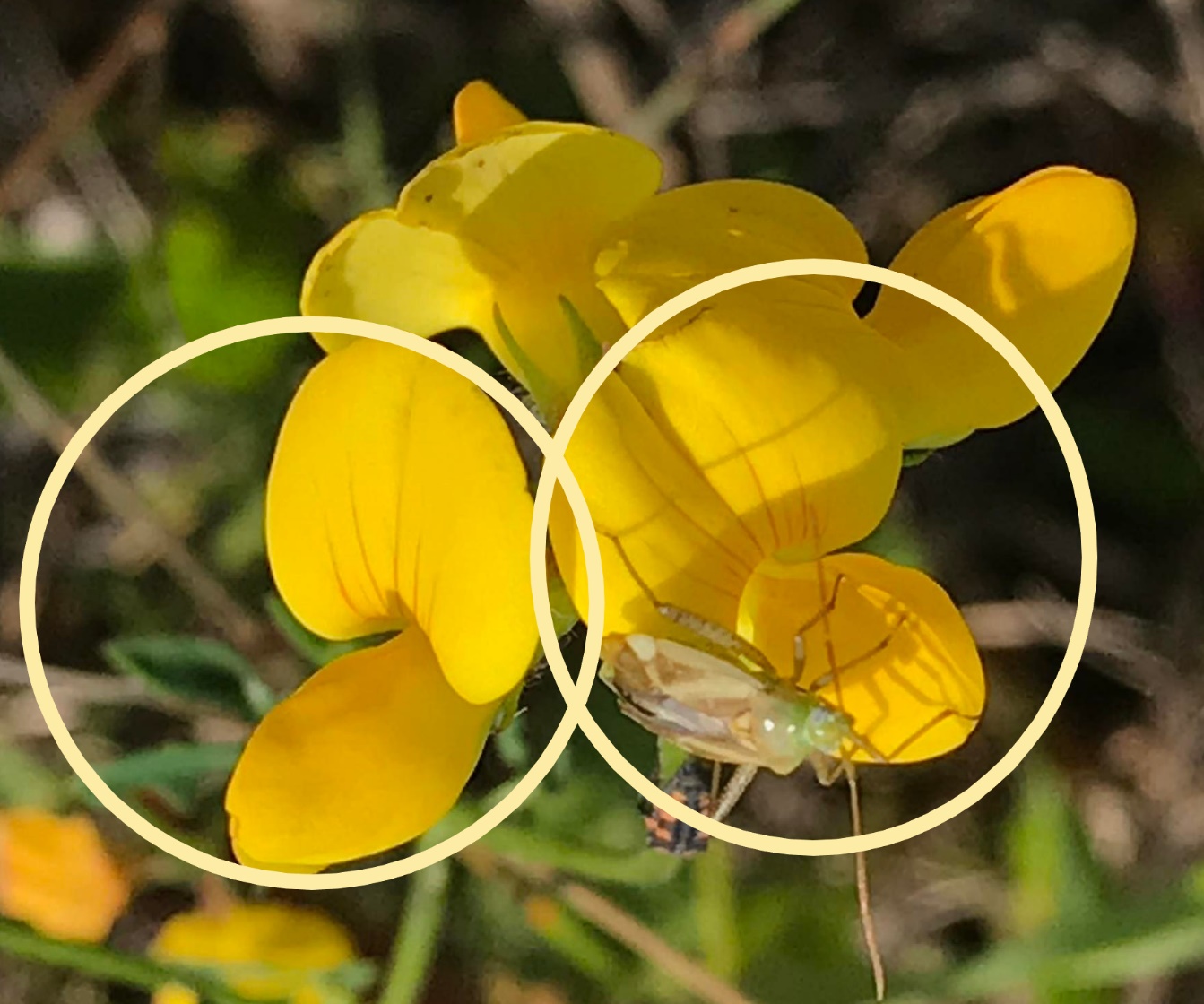


**Supplementary figure 5.** Image of *Lotus corniculatus* in flower with floral units circled (Image credit: Zoe Xirocostas).

***Prunella vulgaris***

For this species we considered one inflorescence as a floral unit. If an invertebrate was to visit multiple flowers within the same floral unit it would count as one visit. If the animal visited several floral units, each would be recorded as a visit.


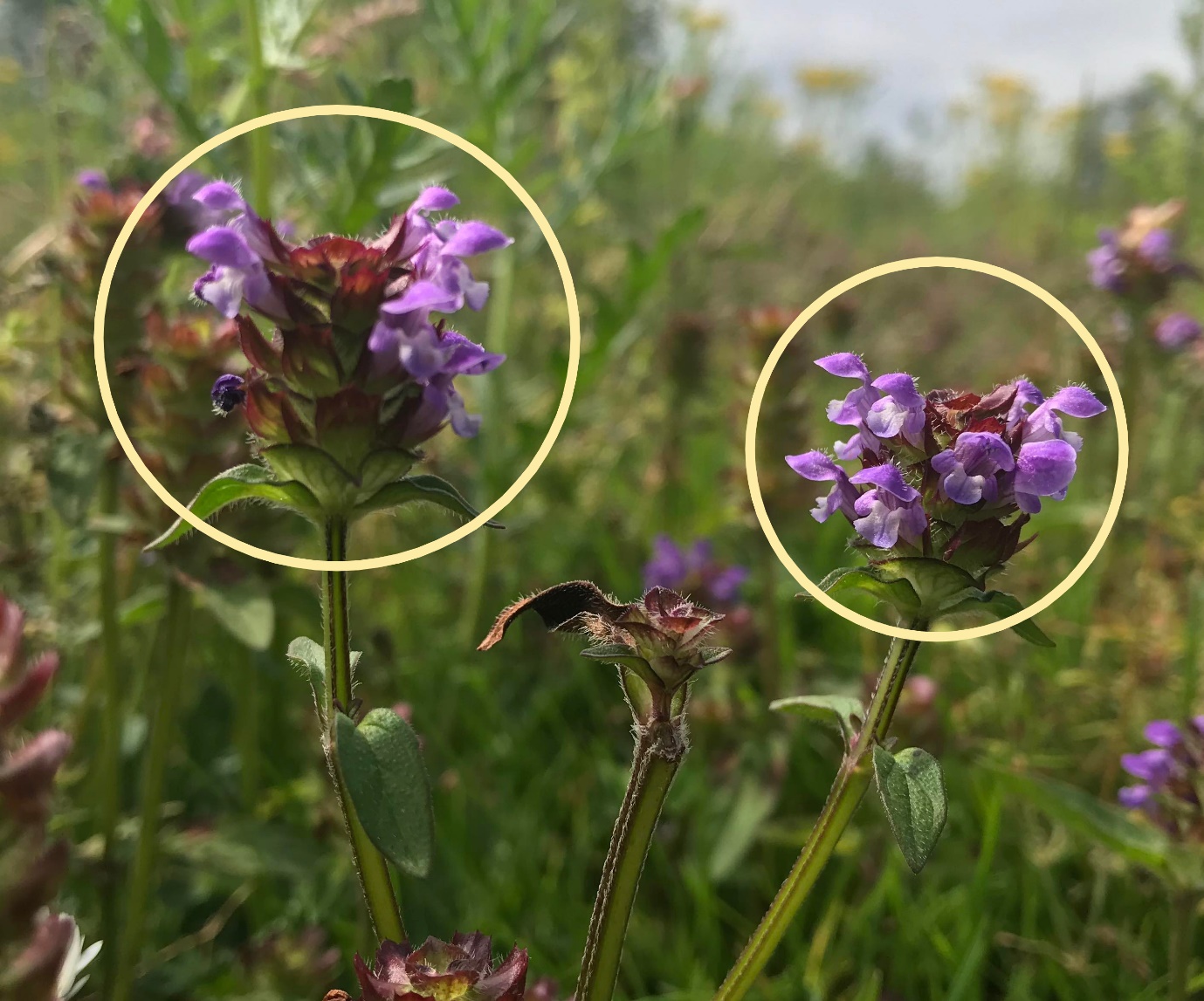


**Supplementary figure 6.** Image of *Prunella vulgaris* in flower with floral units circled (Image credit: Zoe Xirocostas).

***Ranunculus repens***

For this species we considered each flower as a floral unit.


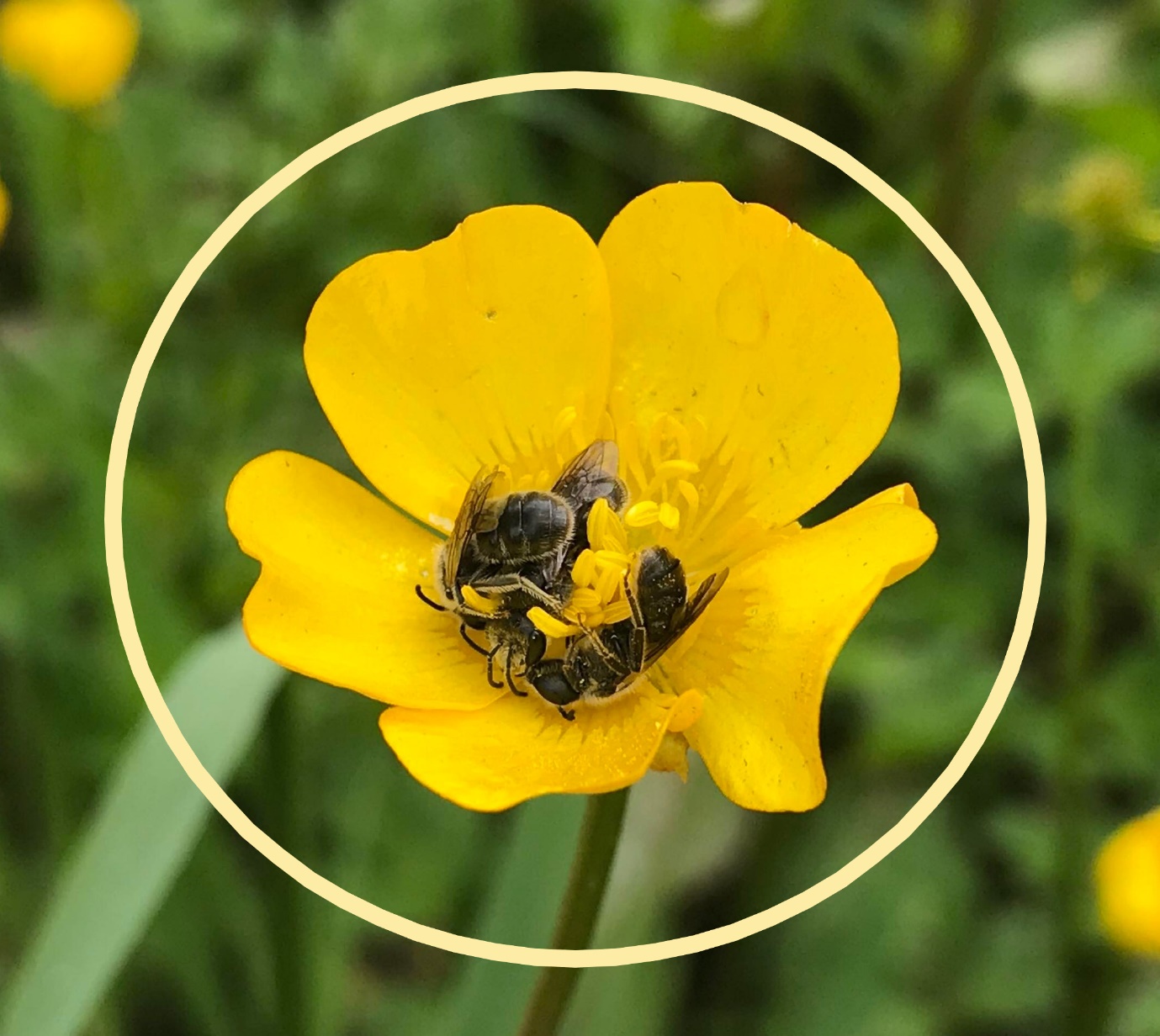


**Supplementary figure 7.** Image of *Ranunculus repens* in flower with floral unit circled (Image credit: Zoe Xirocostas).

***Silene gallica***

For this species we considered each flower as a floral unit.


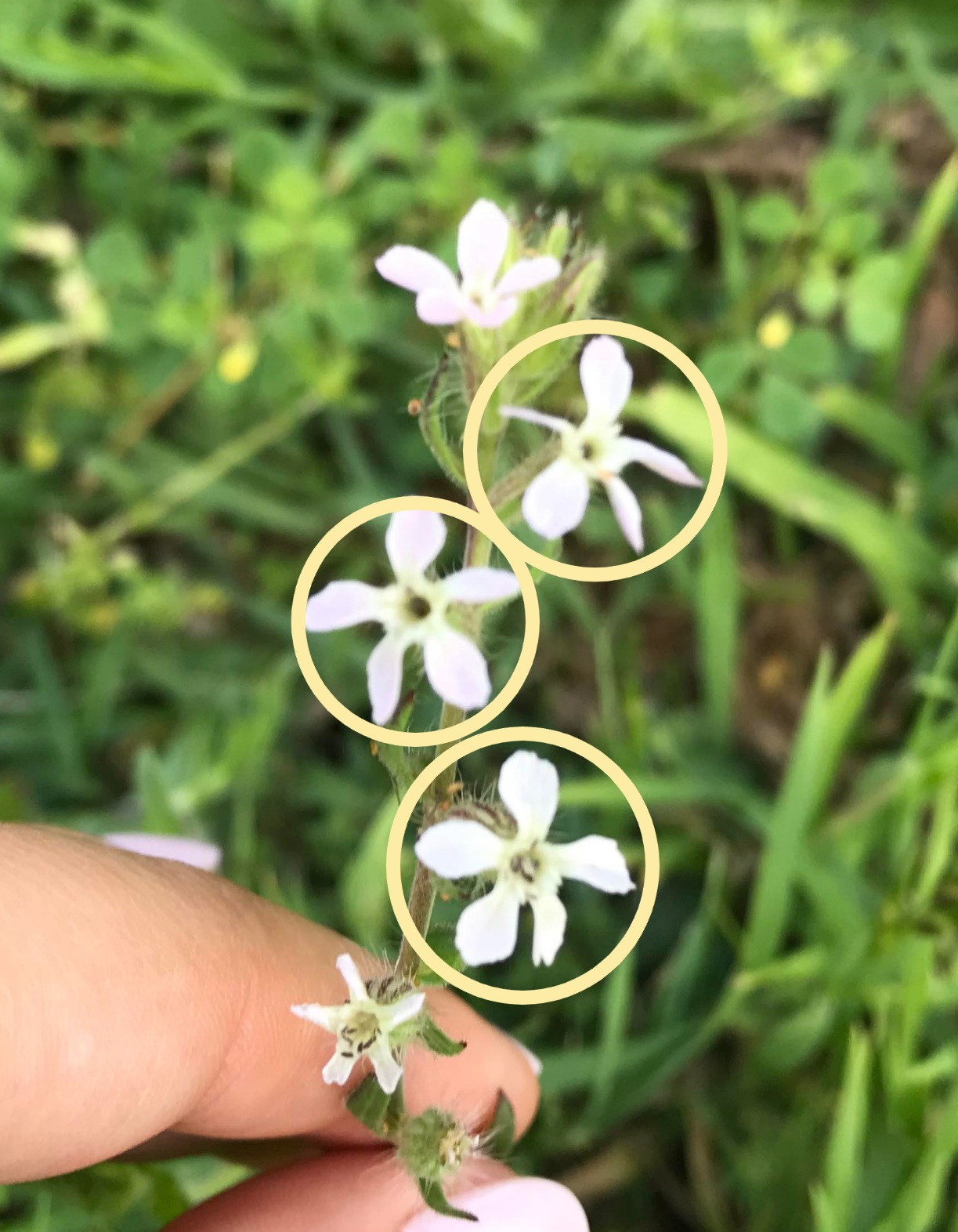


**Supplementary figure 8.** Image of *Silene gallica* in flower with floral units circled (Image credit: Zoe Xirocostas).

***Trifolium repens***

For this species we considered one inflorescence as a floral unit (Fig. S2i). If an invertebrate was to visit multiple flowers within the same floral unit it would count as one visit. If the animal visited several floral units, each would be recorded as a visit.


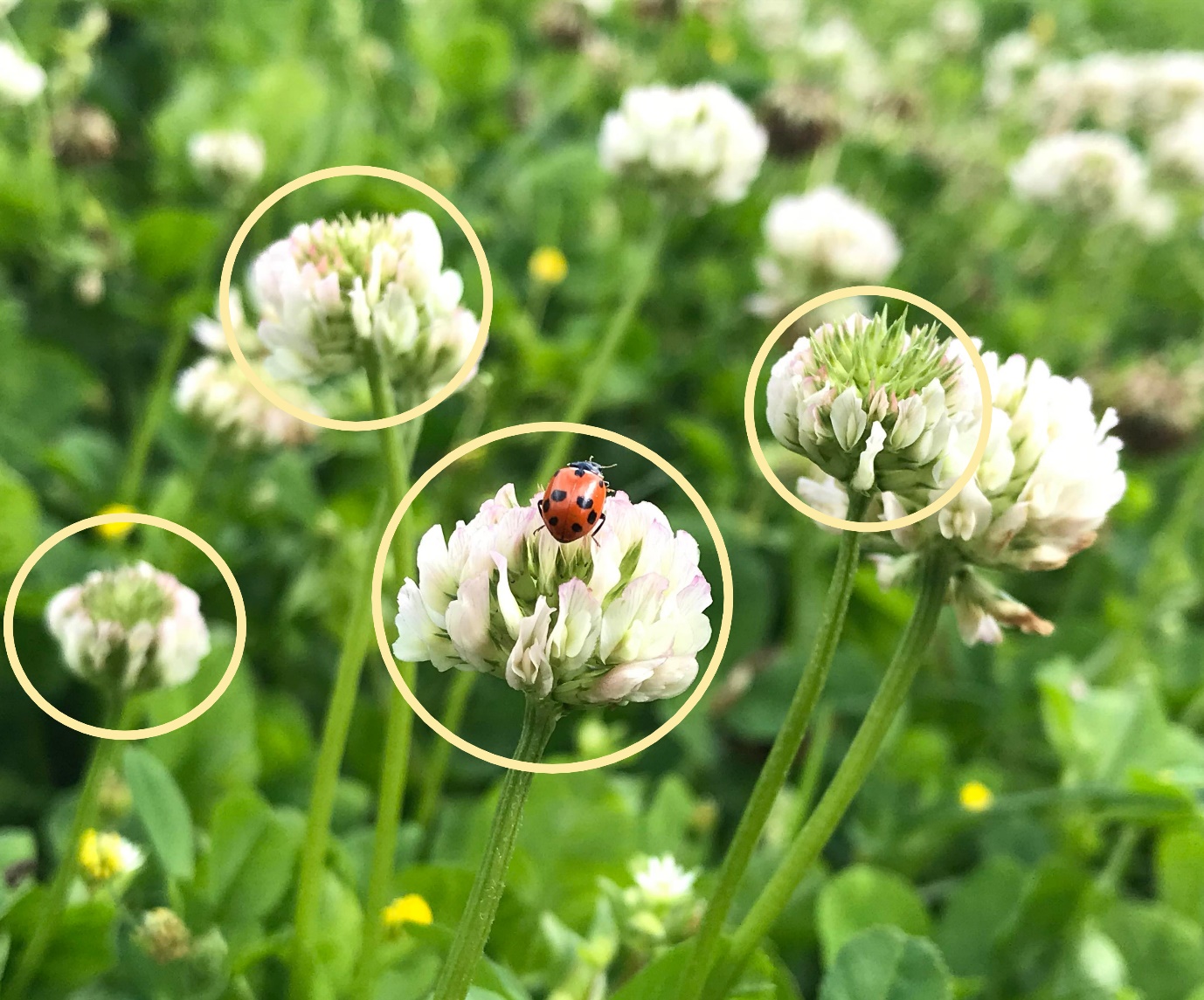


**Supplementary figure 9.** Image of *Trifolium repens* in flower with floral units circled (Image credit: Zoe Xirocostas).

***Verbascum thapsus***

For this species we considered each flower as a floral unit.


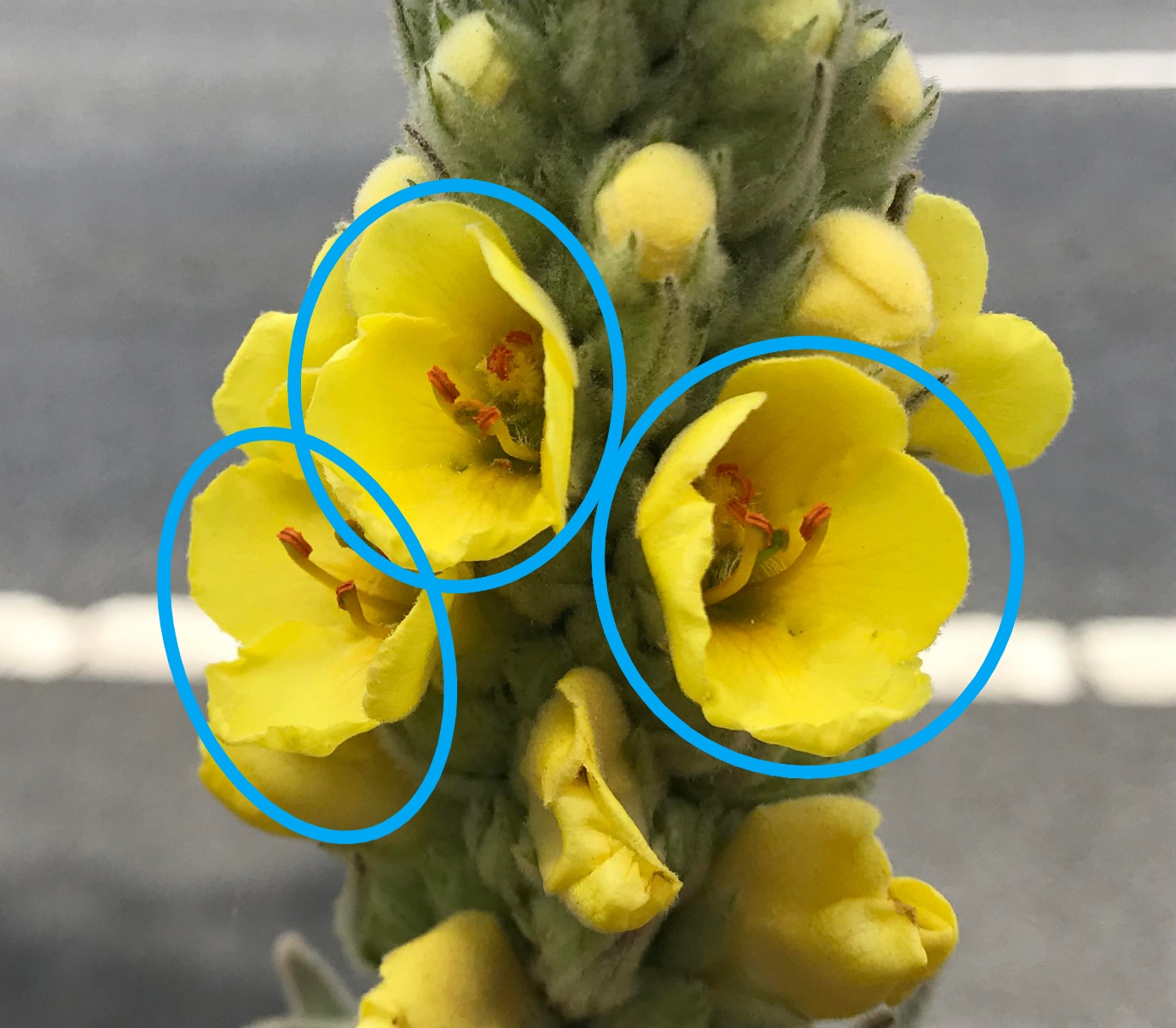


**Supplementary figure 10.** Image of *Verbascum thapsus* in flower with floral units circled (Image credit: Zoe Xirocostas).

**Supplementary figure 11.** Variation in mean visitor abundance per floral unit across sites in the native and introduced ranges for each target species. Bars represent means ± SE.

**Supplementary figure 12.** Variation in mean visitor richness per floral unit across sites in the native and introduced ranges for each target species. Bars represent means ± SE.

**Supplemental information references**

Dwyer, J. (2021). Red valerian (Centranthus ruber [L.] DC ). *Australian Garden History*, *32*(3), 21–23. https://doi.org/10.3316/informit.640601570483368

Holmes, P. M., Rebelo, A. G., & Irlich, U. M. (2018). Invasive potential and management of naturalised ornamentals across an urban environmental gradient with a focus on Centranthus ruber. *Bothalia - African Biodiversity & Conservation*, *48*(1), 1–14. https://doi.org/10.4102/abc.v48i1.2345
